# *Oroxylum indicum* (L.) Bark Ameliorates Anxiety and Depression: Evidence From Experimental and Computational Studies

**DOI:** 10.1002/fsn3.71391

**Published:** 2026-01-14

**Authors:** Md. Aktaruzzaman, Md. Enamul Kabir Talukder, Trina Mitra, Md. Asibur Rahman, Md. Tarikul Islam, Jannatul Ferdous, Nazmul Hossain, Ahmed Saif, Jonas Ivan Nobre Oliveira, Md. Obayed Raihan, Saira Rehman, Bratati Sikdar, Kishore Kumar Sarkar

**Affiliations:** ^1^ Department of Pharmacy, Faculty of Biological Science and Technology Jashore University of Science and Technology Jashore Bangladesh; ^2^ Organic Pharmacy and Medicinal Chemistry Laboratory, Department of Pharmacy Jashore University of Science and Technology Jashore Bangladesh; ^3^ Laboratory of Advanced Computational Biology, Biological Research on the Brain (BRB) Jashore Bangladesh; ^4^ Department of Genetic Engineering and Biotechnology Jashore University of Science and Technology Jashore Bangladesh; ^5^ Department of Pharmacy Jahangirnagar University Savar Dhaka Bangladesh; ^6^ Department of Pharmacy, Faculty of Health Science Gono Bishwabidyalay Savar Bangladesh; ^7^ Department of Pharmacy, Faculty of Science University of Rajshahi Rajshahi Bangladesh; ^8^ Department of Biophysics and Pharmacology, Bioscience Center Federal University of Rio Grande do Norte Natal Rio Grande do Norte Brazil; ^9^ Department of Pharmaceutical Sciences, College of Pharmacy Chicago State University Chicago Illinois USA; ^10^ Faculty of Pharmaceutical Sciences Lahore University of Biological and Applied Sciences Lahore Punjab Pakistan; ^11^ Department of Biological Sciences Bose Institute, Unified Academic Campus Salt Lake, Kolkata West Bengal India

**Keywords:** ADMET, antidepressant, anxiolytic, GC–MS profiling, MD simulations, molecular docking, *Oroxylum indicum*, sedative

## Abstract

This study evaluated the antioxidant and neuropharmacological potentials of methanol extract of *Oroxylum indicum* bark (MOIB) to advocate the scientific basis of its traditional use in medical folklore. The preliminary phytochemical screening and GC–MS analysis identified twenty phytochemicals in MOIB. Besides, in EPM, MOIB exhibited an increase in time spent and the number of entries in open arms while an enhancement in head dipping was demonstrated by MOIB in HBT compared to control, indicating anxiolytic activities. Furthermore, a dose‐dependent reduction in locomotor activities together with immobility time was revealed by MOIB in the hole cross test, open field test, and tail suspension test, forced swimming test, respectively in comparison to that of control suggesting sedative and antidepressant activities. Again, the molecular docking analysis revealed two compounds CID 550198 and CID 10393 with a good binding affinity to all the targeted receptors together with promising pharmacokinetic and toxicity profiles evident from ADME/T analysis. Consequently, the respective molecular dynamic simulation study confirmed the stability of the protein‐ligand complexes. Moreover, two compounds with CID 550198 and CID 10393 might be used as natural lead compounds for the treatment of anxiety, sleep disorders, and depression. Yet, advanced studies are required to reveal the fundamental mechanism of these activities.

## Graphical abstract

The study evaluated the antioxidant and neuropharmacological effects of Oroxylum indicum bark (MOIB), identifying 20 phytochemicals through GC–MS analysis. MOIB exhibited anxiolytic, sedative, and antidepressant effects in various behavioral tests, with two compounds (CID 550198 and CID 10393) showing strong binding affinities to targeted receptors and promising pharmacokinetic profiles. Molecular dynamic simulations confirmed the stability of these complexes, suggesting these compounds as potential treatments for anxiety, sleep disorders, and depression.

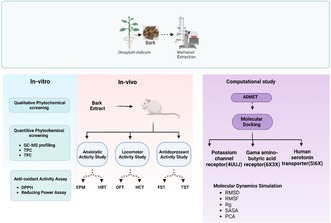

**Abbreviations**
ADME: Absorption, distribution, metabolism, excretion
DPPH: 2,2‐diphenyl‐1‐picrylhydrazyl
EPM: Elevated plus maze test
FST: Forced swimming test
HBT: Hole board test
HCT: Hole cross test
MD simulation: Molecular dynamic simulation
MOIB: Methanol extract of *Oroxylum indicum* bark
OFT: Open field test
ROS: Reactive oxygen species
TFC: Total flavonoid content
TPC: Total Phenolic content
TST: Tail suspension test

## 1 Introduction

Depression and anxiety are the most common mental illnesses that people experience once or several times during their lifespan. They not only affect daily activities but also significantly affect life expectancy (Mahendran and Vijayan 2018). The World Health Organization has recognized anxiety and depressive disorders as the leading cause of non‐fatal health suffering (Adnan, Chy, Kamal, et al. 2020). The phenomenon of depression is a multiplex, persistent mental disorder with a large impact on the community, which is often associated with functional impairment and an increased risk of mortality and morbidity; in contrast, anxiety refers to a state of unease, such as worry or fear (Möller et al. 2016). It is still unclear what causes depression and anxiety, but they may be related to an imbalance in the antioxidant defense system that leads to oxidative stress or redox imbalance. When excess oxidative reactive species (ROS) are produced in the brain, cellular discrepancy occurs, which leads to cognitive decline and neurological damage (Adnan, Chy, Mostafa Kamal, et al. 2020). Several studies have reported that patients with extreme depression had elevated ROS levels in their plasma and brain, leading to cognitive degeneration and neurodegenerative diseases (Ali, Al, et al. 2021; Islam et al. 2021). Moreover, the malfunction of the antioxidant defense system causes redox inequity, or oxidative stress, which results in an excessive generation of ROS in the brain as a consequence of cellular disparity and consequently leads to cognitive impairment as well as damage to neurobiological mechanisms (Ali, Al, et al. 2021). Consumption of antioxidants may reduce the amount of ROS generated in the central nervous system and therefore improve continuing tissue damage, but a single antioxidant therapy may not provide the desired results. Hence, as a part of pharmacotherapy and polypharmacy, continuous administration of antipsychotic drugs and antioxidant‐rich dietary supplements is recommended to manage these life‐threatening diseases that lead patients to aversion to receiving medicines (Adnan, Chy, Kamal, et al. 2020). However, there are so many prescribed antidepressant drugs, including selective serotonin, benzodiazepines, and serotonin‐norepinephrine reuptake inhibitors, which are not very effective in controlling depression, anxiety, oxidative stress, and chronic inflammation due to their undesirable side effects such as sexual dysfunction, sedation, memory disturbances, amnesia, abuse liability, and daytime sleepiness. In these circumstances, global research attention is focused on potential plant‐derived bioactive phytochemicals with fewer side effects (Adnan, Chy, Kamal, et al. 2020). Various medicinal plants are traditionally used to prevent disease as they deliver a plethora of phytoconstituents and thus contribute to developing drug candidates, and a variety of phytochemicals can be extracted from medicinal herbs, fruits, and vegetables and may mitigate neurodegeneration and enhance memory and cognitive function (Saha et al. 2013; Cox and American 1994; Sarkar, Islam, et al. 2021; Anisuzzman et al. 2017; Sarkar et al. 2022; Sarkar et al. 2024).

*Oroxylum indicum* is widespread throughout Asian countries, including Thailand, India, Vietnam, Malaysia, the Philippines, Indonesia, China, Japan, and Taiwan (Dev et al. 2010). It belongs to the family Bignoniaceae and grows naturally in Chittagong and Sylhet–two hilly regions of Bangladesh. A variety of parts of this plant have traditionally been used to treat diabetes, biliousness, cancer, inflammation, skin disorders, and rheumatoid arthritis (Jagetia 2021). Moreover, several phytochemicals are reported for the steam bark of *O. indicum* , including 5,7‐dihydroxy‐3‐methoxyflavone, 5,7‐dihydroxyflavone, 3,5,7‐trihydroxyflavone, 3,5,7,4′‐tetrahydroxyflavone, 5,7,4′‐trihydroxy‐3‐methoxyflavone, and 5,7,4‐trihydroxyflavone, and the structures of these chemicals have also been characterized (Bahadur Chetry and Bharali 2018; Begum et al. 2019; Halevas et al. 2021; Ramírez et al. 2025). Yet, the literature review shows only a very limited number of studies have been conducted on *O. indicum* . Even though the plant has many traditional uses, there have not been any exclusive scientific investigations conducted to investigate the antioxidant and neuropharmacological effects of this plant using experimental and in silico methods. Therefore, the objective of this study was to systematically investigate the antioxidant, anxiolytic, and antidepressant activities of *O. indicum* bark through experimental and in silico methods to identify the natural bioactive compounds, followed by determining the potential lead constituents from MOIB.

## 2 Materials and Methods

### 2.1 Chemicals and Reagents

Several analytical‐grade chemicals and reagents were used in these experiments, including methanol, gallic acid, quercetin, acetic acid, folin–ciocalteu reagent (FC), and 2,2‐Diphenyl‐1‐picrylhydrazyl (DPPH), all procured from Merck, Germany. Besides, imipramine hydrochloride and diazepam were purchased from Square Pharmaceuticals Ltd., Bangladesh.

### 2.2 Identification and Collection of Plant Material

*Oroxylum indicum* bark was collected from the Khagrachari Hill Tract, Chittagong, Bangladesh, in January 2021. For future reference, a voucher specimen (DACB: 90632) was deposited at the Bangladesh National Herbarium authenticated by Khandakar Kamrul Islam, Senior Scientific Officer of the Bangladesh National Herbarium.

### 2.3 Extraction

The barks were not only washed with distilled water for a single time but also washed with running water three to four consecutive times, and shade drying was allowed for 2–3 weeks to prevent unwanted materials. To obtain fine powder, the dried samples were ground into coarse powder using a laboratory grinding mill (Model 2000 LAB Eriez) and passed through a 40‐mesh sieve. Following this, 300 g of powder were soaked in 1200 mL of methanol for 14 days with constant stirring. Using a cotton plug and Whatman Grade 1 filter paper (Sigma‐Aldrich, St. Louis, MO, USA), the mixture was filtered. Lastly, the crude extract (2.51% w/w) was obtained using a rotary evaporator (RE‐100 PRO, DLAB Scientific Inc., Beijing, China).

### 2.4 Experimental Animals

We purchased both male and female *Swiss albino* mice weighing 20–25 g with 6 weeks of age from the International Center for Diarrheal Disease and Research, Bangladesh (ICDDR, B). Mice were then acclimatized to the laboratory environment (24°C temperature, 50%–70% relative humidity, and 12 h of light and darkness). A typical diet and distilled water were also provided to the experimental rodents. Moreover, the Ethical Review Committee of the Faculty of Biological Science and Technology, Jashore University of Science and Technology approved all the experiments that were performed as per the recommended guidelines of this committee [Ref: ERC/FBST/JUST/2021–81].

### 2.5 Qualitative Phytochemical Screening

A preliminary qualitative phytochemical study was conducted on MOIB using standard methods described by Kebede et al. (2021).

### 2.6 GS‐MS‐Based Phytochemicals Identification

A Clarus 690 gas chromatograph (PerkinElmer, CA, MA, USA) was used for gas chromatography–mass spectrometry analysis, which was equipped with a column (Elite‐35, 30 m × 0.25 mm; PerkinElmer, CA, MA, USA) and 0.25 m film along with a mass spectrophotometer (Claus SQ 8 C; PerkinElmer, CA, MA, USA). Using pure helium (99.999%) as the carrier gas with a constant flow rate of 1 mL/min, the 1 μL sample was injected in a splitless manner, and the time was allowed for 40 min, and the sample analysis was conducted using electron ionization mode at 70 eV energy. Even though the inlet temperature remained constant at 280°C, the column oven temperature was fixed at 60°C for 0 min; the temperature was then increased by 5 degrees per minute to 240°C, which lasted over 4 min. Besides, scan time and mass range were 1 s and 50–600 m/z, respectively. Then phytochemicals were identified by comparing them to the NIST database (Rahman et al. 2021).

### 2.7 Assessment of In Vitro Antioxidant Activity

#### 2.7.1 Total Phenolic Content Assay (TPC)

MOIB was analyzed using folin–ciocalteu reagent (FC) to determine its total phenolic content (TPC), as reported by Sarkar et al. (2022) and Medha et al. (2022). As part of this assay, a standard curve for gallic acid was prepared using various concentrations, ranging from 100 to 500 μg/mL. Initially, 9 mL of distilled water, along with 1 mL of FC reagent (10% v/v), was combined with 1 mL of solution taken from each concentration into volumetric flasks. Following 5 min, distilled water was added with 10 mL of a 7% w/v sodium carbonate solution to adjust the volume to 25 mL. After incubation for 30 min, absorbance was measured at 750 nm using a blank solution. Results were expressed as mg gallic acid equivalent per gram of extract (mg GAE/g).

#### 2.7.2 Total Flavonoid Content Assay (TFC)

The total flavonoid content of MOIB was determined using a standard quercetin calibration curve (Sarkar et al. 2022). For the purpose of building the standard calibration curve of quercetin, different concentrations ranging from 100 to 500 μg/mL were used. In volumetric flasks, 1 mL of quercetin solution from each concentration was added with 4 mL of distilled water, followed by 0.3 mL of sodium nitrous solution (5% w/v). Once the mixture had been incubated for 5 min, 0.3 mL of aluminum chloride (10% w/v) was added to it. Then, a final volume of 10 mL was adjusted after adding 2 mL of sodium hydroxide (1 M). In the following step, the absorbance at 510 nm was measured against a blank, and the results were expressed as mg quercetin equivalent (QE) per g of extract (mg QE/g).

#### 2.7.3 DPPH Free Radical Scavenging Assay

The DPPH scavenging assay was conducted to determine the antioxidant potential of MOIB following the method described by Zilani et al. (2016). Here, various concentrations (between 1024 and 2 μg/mL) of both standard and extract were made according to the serial dilution method. Then, 6 mL of alcoholic DPPH solution (0.004% w/v) was combined with 2 mL of standard and extract solutions from each concentration. Using ascorbic acid as a standard, the absorbance at 517 nm was measured after 30 min of incubation following:

$$\text{Percent Scavenging}=\left[1-\left(\mathrm{Absorbance}\;\mathrm{of}\;\text{Sample}/\text{Standard}/\mathrm{Absorbance}\;\mathrm{of}\;\text{Control}\right)\right]\times 100.$$

#### 2.7.4 Reducing Power Assay

The reducing power assay was conducted according to the method described by Sarkar et al. (2022). In this study, various standard and extract concentrations (100, 200, 300, 400, and 500 μg/mL) were prepared. Next, 2.5 mL of phosphate buffer (200 mmol/L, pH 6.6) and 2.5 mL of potassium ferricyanide (1% w/v) were mixed with 1 mL of an aliquot from each concentration. Subsequently, 20 min were allowed for the incubation of the mixture, and then, 2.5 mL of 10% (w/v) trichloroacetic acid was combined with it. After that, 2.5 mL of supernatant was taken from the mixture after centrifugation at 3000 rpm for 10 min and blended with 0.5 mL of ferric chloride (0.1% w/v), where continuous shaking was maintained. At last, 5 min later, the absorbance was recorded at 700 nm in contrast to a blank solution.

### 2.8 Experimental Design

The experimental animals were randomly divided into four groups (control, standard, and two test groups), each containing five *Swiss albino* mice. The control group was treated with vehicle (1% Tween 80 in distilled water, 10 mL/kg) and the standard group received diazepam (1 mg/kg, p.o.) for elevated plus maze tests, hole board tests, open field test, and hole cross test, whereas in the case of tail suspension and forced swimming tests, imipramine hydrochloride (10 mg/kg, p.o.) was administered as a standard drug. Moreover, MOIB at doses of 200 and 400 mg/kg, b.w., was administered to the test groups through the oral route.

### 2.9 Acute Toxicity Study

*Swiss albino* mice aged around 6‐7 weeks were used in an oral acute toxicity study of MOIB following guidelines provided by the OECD (Organization for Economic Cooperation and Development). After fasting overnight, three male and three female mice were administered a single high dose of MOIB (5000 mg/kg body weight), whereas a control group containing both three male and female mice was given normal saline. After 1 h of the treatment, the mice were fed, and a variety of careful observations, including changes in skin, lethargy, coma, diarrhea, and salivation, were checked to determine any sign of toxicity, which were continued for 14 days (Sarkar et al. 2022).

### 2.10 Anxiolytic Activity Study

#### 2.10.1 Elevated Plus Maze Test (EPM)

We assessed the anxiolytic activity of MOIB using the method described in Sarkar, Rahman, et al. (2021) and Sarkar, Islam, et al. (2021), with minor modifications. The EPM apparatus consisted of two opposing open arms measuring 50 cm × 10 cm × 70 cm and two opposing closed arms measuring 50 cm × 10 cm × 30 cm. The two arms were raised 40 cm from the ground and displayed the plus sign symbol by merging on a central platform (5 cm × 5 cm). After 30 min of the treatment with MOIB at 200 and 400 mg/kg body weight (p.o.) to the test group mice and diazepam at 1 mg/kg (p.o.) to the standard group and vehicle at 10 mL/kg (1% Tween 80 dissolved in water) to the control group, each mouse from the respective group was placed in the center of the platform and subsequently, the number of entries along with the amount of time spent in the open arms was measured for 5 min (Goni et al. 2020).

#### 2.10.2 Hole Board Test (HBT)

This test was conducted under the procedure described by Sonavane et al. (2002). In this experiment, a wooden board with 16 equally distributed holes with 3.3 and 2.2 cm in diameter and depth, respectively, was utilized. MOIB was administered orally at doses of 200 and 400 mg/kg body weight (b.w.) to the sample groups of mice as described above. Besides, the control group received vehicle (1% Tween 80 in distilled water, 10 mL/kg), while the standard group mice were administered diazepam (1 mg/kg, p.o.). After 30 min of treatment, an individual mouse from different groups was kept in a corner of a wooden board, and then, the number of heads dipping into the holes was recorded for 5 min at 0, 30, 60, 90, and 120 min (Goni et al. 2020).

### 2.11 Locomotor Activity Study

#### 2.11.1 Open Field Test (OFT)

The open‐field test was conducted following the method described by Leaves et al. (2021). Here, the apparatus is divided into sixteen squares (76 cm × 76 cm) with alternating black and white colors and is composed of a wall (40 cm high) which gives a chess board‐like pattern. Mice grouping and sample administration were accomplished as like as the hole board test. Following 30 min of treatment, each mouse was placed in the middle of the apparatus, and afterward, the number of squares crossed was measured at 0, 30, 60, 90, and 120 min for 3 min, where all mice of the different groups were tested in a quiet and noise‐free environment (Chowdhury et al. 2020).

#### 2.11.2 Hole Cross Test (HCT)

This experiment was conducted following the method described by Apu et al. (2012) with minor modifications. The test apparatus is a wooden box with dimensions of 30 cm × 20 cm × 14 cm and a partitioning wall that contains a hole of 3 cm in diameter at a height of 7.5 cm from the floor. Following the administration of the test samples, the spontaneous movement (number of crossings) of individual mice across the opening was recorded for 3 min from one compartment to another at 0, 30, 60, 120, and 180 min, respectively (Islam et al. 2021).

### 2.12 Antidepressant Activity Study

#### 2.12.1 Forced Swimming Test (FST)

To evaluate MOIB's antidepressant effects, the forced swimming test was conducted using the method described by Alves et al. (2020). The investigational swimming apparatus consisted of a transparent glass tank with a dimension of 25 cm × 15 cm × 25 cm, which was loaded with 15 cm of water at (25 ± 1)°C. To familiarize the animals with the experimental system, an introductory test was conducted the day before the final experiment. We placed individual mice in the tank for 6 min after 30 min of treatment in the test groups (200 and 400 mg/kg b.w., p.o.), in the standard group (imipramine hydrochloride, 10 mg/kg b.w., p.o.) and in the control group (1% Tween 80, 10 mL/kg, p.o.), where the first couple of minutes were considered as initial adjustment time and the next 4 min for the measurement of immobility period.

#### 2.12.2 Tail Suspension Test (TST)

This test was performed according to the method described by Ali, Al, et al. (2021). In this test, mice were suspended by the tail with an adhesive tape about 1 cm from the tip of the tail to achieve immobility after 30 min of oral administration of 1% Tween 80, 10 mL/kg, and MOIB 200 and 400 mg/kg to the control and test groups, respectively followed by imipramine hydrochloride, 10 mg/kg to the standard group. Over the 6‐min observation period, the first 2 min were considered as the initial adjusting period, and the last 4 min were for the calculation of total immobile time.

### 2.13 Statistical Analysis

IBM SPSS Statistics 23 was used for the statistical analysis. The data were analyzed using one‐way analysis of variance (ANOVA) and Dunnett's test. The test results were presented as the mean ± SEM (standard error of the mean), and p*** < 0.001, p** < 0.01, and p* < 0.05 exhibited statistical significance compared to the control group.

### 2.14 In Silico Study

#### 2.14.1 ADME Analysis

In the drug development process, the study of pharmacokinetic parameters (absorption, distribution, metabolism, and excretion) is highly significant (Daina et al. 2017). Here, SwissADME (http://www.swissadme.ch/index.php), a web‐based server, was utilized for a better understanding of pharmacokinetic properties, where some influential factors, including oral bioavailability, GI absorption, BBB permeability, Lipinski's rules, Log *p* ≤ 5, MW < 500 amu, HBD sites < 5, nRB ≤ 10, TPSA ≤ 140, and HBA < 10, were taken into account (Lipinski et al. 2012; Hsiao et al. 2021; Veber et al. 2002).

#### 2.14.2 Toxicity Analysis

ProTox‐II (https://tox‐new.charite.de/) and AdmetSAR (http://lmmd.ecust.edu.cn/admetsar2/) were used to evaluate the toxicological effects of the identified phytochemicals. Several parameters, including Ames toxicity, hepatotoxicity, immunogenicity, mutagenicity, carcinogenicity, and LD_50_, were deliberated in this analysis (Hsiao et al. 2021; Cheng et al. 2012).

#### 2.14.3 Ligand Preparation

Based on the ADMET analysis, 7 compounds were selected from the GC–MS analysis of MOIB. Then, the 3D structure of those compounds, along with control drugs (diazepam and imipramine HCL) were downloaded from the database (http://www.mcbi.nlm.nih.gov/) in SDF format. The LigPrep option of Maestro v11.4. was used to refine, process, and prepare ligands (Friesner et al. 2006; Talukder et al. 2025). Finally, the chemicals were adjusted by using the OPLS3e force field for a molecular docking study (Harder et al. 2016).

#### 2.14.4 Protein Preparation

The 3D structures of the human serotonin transporter (PDB ID: 5I6X) (Coleman et al. 2016), potassium channel receptor (PDB ID: 4UUJ) (Lenaeus et al. 2015), and GABA_A_ receptor (PDB ID: 6X3X) (Kim et al. 2020) were retrieved from the Protein Data Bank (https://www.rcsb.org/) in PDB format. 4UUJ, 5I6X, and 6X3X had 111, 549, and 358 amino acid sequences, respectively. Unnecessary chains, metals, water, and co‐factors were deleted using the protein preparation wizard of the Schrodinger suite 2020–3 under a Linux environment (Bowers et al. 2006; Madhavi Sastry et al. 2013). Besides, the OPLS3e force field was used to optimize the protein structures (Harder et al. 2016). Next, to conduct a molecular docking study, the chains C, A, and D were utilized for the potassium channel receptor (PDB ID: 4UUJ), human serotonin transporter (PDB ID: 5I6X), and GABA_A_ receptor, respectively.

#### 2.14.5 Grid Generation and Molecular Docking Analysis

Molecular docking was executed to determine the binding affinity of the selected compounds to the targeted receptors associated with anxiolytic, antidepressant, and sedative activities (Islam et al. 2024; Talukder et al. 2024). Selected 7 compounds, including the standards, were docked using the Glide package of the Schrödinger Suite with the desired macromolecule. OPLS3e was utilized as a force field in standard precision mode during docking (Friesner et al. 2006). The grid generation for the targeted receptors was completed using the native inhibitors (N3) in a complex with the desired proteins to determine the binding position. In addition, a receptor grid with box diameter *X* = 30, *Y* = −30, and *Z* = 30 was made for the potassium channel receptor (4UUJ) and in the case of the human serotonin transporter (5I6X), and the human gamma‐aminobutyric acid GABAA receptor (6X3X), the box diameters were *X* = 30, *Y* = −30, and *Z* = 30 and *X* = 30, *Y* = −30, and *Z* = 30, respectively. Then, Maestro Viewer was utilized to visualize residues bound to ligands along with different types of chemical bonds.

#### 2.14.6 Molecular Dynamics (MD) Simulation

A 100 ns molecular dynamics simulation was run to determine the binding equilibrium of protein‐ligand complexes using the Desmond package accessible in the Schrödinger suite (Academic Version) under a Linux environment (Bharadwaj et al. 2021). Initially, prepared protein‐ligand complexes from molecular docking studies were further utilized for MD simulation studies (Goyal and Goyal 2020). To maintain a specific volume, an orthorhombic periodic boundary box was added to the simple point‐charge water model (SPC). Na^+^ and Cl^−^ ions were added to the solvated environment to sustain a salt concentration of 0.15 M. The OPLS3e force field was used to minimize and relax the environment (Roos et al. 2019). An ensemble of constant pressure‐constant temperature (NPT) with 1.01325 bar pressure and 300.0 K temperature was maintained.

#### 2.14.7 Simulation Trajectory Analysis

Schrodinger's Maestro interface version 9.5 was utilized to render all molecular dynamics simulation snapshots. A simulation interaction diagram (SID) was set to calculate the simulation event generated by the MD simulation in the Schrödinger package. Using the trajectory output, the root‐mean‐square deviation (RMSD), root‐mean‐square fluctuation (RMSF), radius of gyration (RG), solvent accessible surface area (SASA), and PCA were calculated.

#### 2.14.8 PCA—Method

Principal Component Analysis (PCA) serves as a computational method utilized to extract and analyze predominant motion patterns from a collection of trajectory data. PCA provides a means to simplify and gain insights into the intricate motions that biomolecules undergo during simulations. The covariance matrix of the structural data was evaluated, and subsequently, the eigenvalues and eigenvectors of this matrix were computed (Alom et al. 2023). This process aimed to capture significant structural deviations by arranging the eigenvectors based on their corresponding eigenvalues in descending order. The first principal component (PC) captured the largest variance within the data, while the second PC encapsulated the second‐largest variance orthogonal to the first one. The PCA computation was executed using SciPy v1.11 within the Python environment v3.11.

## 3 Results

### 3.1 Preliminary Phytochemical Screening

The phytochemical screening results confirmed the presence of a variety of bioactive compounds, namely tannins, glycosides, coumarins, saponins, and flavonoids; in contrast, alkaloids, steroids, and carbohydrates were found to be absent, which was similar to the previous studies, as listed in Table 1.

**TABLE 1:** Preliminary phytochemical screening of MOIB.

Serial no.	Secondary metabolites	Phytochemical test	MOIB
1.	Alkaloids	Mayer's test	−
2.	Tannins	Lead acetate test	+
3.	Glycoside	Foam test	+
4.	Steroids	Salkowski's test	−
5.	Carbohydrates	Fehling's test	−
6.	Coumarins	Alkaline reagent test	+
7.	Saponins	Frothing and foaming test	+
8.	Flavonoids	Alkaline reagent test	+
9.	Triterpenes	Salkowski Test	+

*Note:* Here, (+) indicates present, and (−) indicates absent.

### 3.2 GS‐MS‐Based Phytochemicals Identification

**FIGURE 1:**
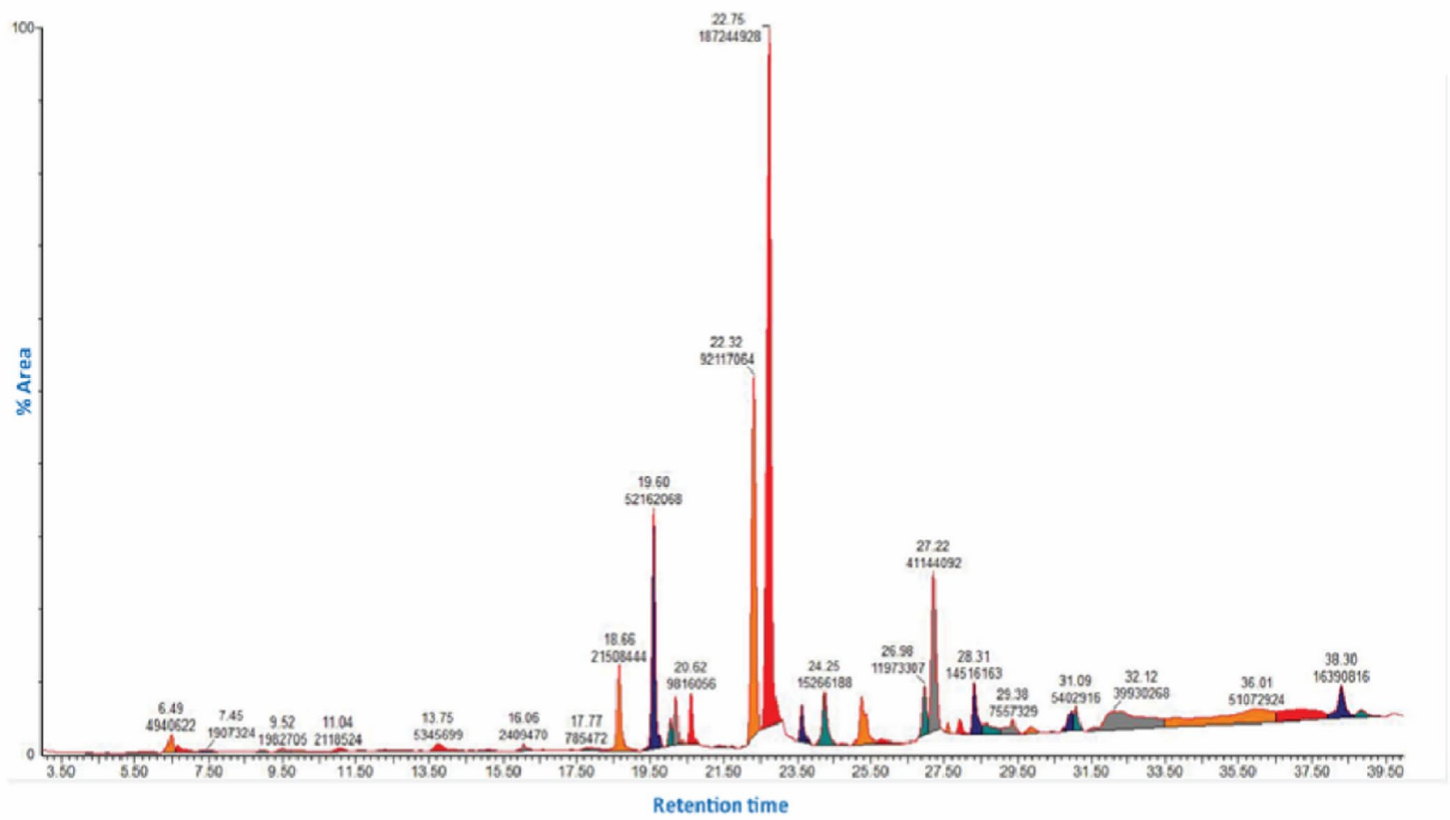
GC–MS chromatogram of methanol extract of *Oroxylum indicum* bark.

GC–MS analysis uncovered twenty bioactive compounds, which were represented by their chemical formula, molecular weight (MW), retention time (RT), % area, CID, nature, and biological activity (Table 2). Additionally, Figure 1 illustrates the total ionic chromatogram (TIC) of MOIB.

**TABLE 2:** Identified compounds from GC–MS analysis.

Sl. no.	Name	Chemical formula	MW	RT	% Area	CID	Nature of compounds	Biological activity	References
1.	2,4,4,6,6,8,8‐Heptamethyl‐1‐nonene	C_16_H_32_	224	18.665	3.05631	545,785	Alkene	Antioxidant activity	Khan et al. (2016)
2.	1,2,3,4‐Tetramethylcyclohexane	C_10_H_20_	140	19.603	7.412132	94,277	Alkane	Not found	
3.	Benzeneethanol, 4‐hydroxy—	C_8_H_10_O_2_	138	20.052	0.707233	10,393	Alcohol	Insecticide	Li et al. (2018)
4.	Eicosen‐1‐ol, cis‐9—	C_20_H_40_O	296	20.206	1.327305	5,364,523	Alcohol	Not found	
5.	9‐octadecenoic acid, (e)‐/trans‐9‐octadecenoic acid	C_18_H_34_O_2_	282	20.615	1.394843	637,517	Fatty acid ester	Liver cancer	Ide et al. (1987)
6.	2‐Methyl‐5‐undecanol	C_12_H_26_O	186	22.324	13.08966	544,080	Alcohol	Anticancer	Cai et al. (2021)
7.	Ethyl 9‐hexadecenoate	C_18_H_34_O_2_	282	22.747	26.60715	5,364,759	Fatty acid ester	Antimicrobial	Sultana et al. (2020)
8.	Carbonic acid, decyl 2‐ethylhexyl ester	C_19_H_38_O_3_	314	23.618	1.283564	91,693,126	Ester	Not found	
9.	Dicyclomine	C_19_H_35_O_2_N	309	24.248	2.169297	3042		Antidiarrheal	Page and Dirnberger (1981)
10.	Clofexamide	C_14_H_21_O_2_N_2_Cl	284	25.247	2.62242	28,554	Amides	Antiviral	Abrams et al. (2020)
11.	Ethyl 10‐undecenoate	C_13_H_24_O_2_	212	26.976	1.701385	12,729	Fatty acid ester	Not found	
12.	1,7‐Dimethyl‐4,10‐dioxa‐1,7‐diazacyclododecane	C_10_H_22_O_2_N_2_	202	27.218	5.846498	560,333	Alkane	Analgesic and Anti‐Inflammatory	Sajeesh and Parimelazhagan (2014)
13.	4‐(3‐Hydroxyphenyl)‐4‐oxobutanoic acid	C_10_H_10_O_4_	194	27.607	0.200219	270,396	Acid	Leukotriene D4 antagonist	Musser et al. (1987)
14.	1,2,3,4 Tetramethylcyclohexane	C_10_H_20_	140	27.928	0.476161	94,277	Alkane	Not found	
15.	l‐(+)‐Ascorbic acid 2,6‐dihexadecanoate	C_38_H_68_O_8_	652	28.31	2.06272	54,722,209	Acid	Anticancer	Begum et al. (2017)
16.	Ethyl 2,4,6‐trimethylbenzoate	C_12_H_16_O_2_	192	29.376	1.073882	74,465	Acid	Not found	
17.	Cholestan‐3‐ol, 2‐methylene‐, (3.beta., 5.alpha.)	C_28_H_48_O	400	29.872	0.447492	22,213,932	Alcohol, Alkene	Antidiabetic	Sheweita et al. (2020)
18.	Heptadecanoic acid, 16‐methyl‐, methyl ester	C_19_H_38_O_2_	298	30.965	0.965916	110,444	Fatty acid	Not found	
19.	Methyl 10‐trans,12‐cis‐octadecadienoate	C_19_H_34_O_2_	294	31.085	0.767744	5,471,014	Linoleic Acid		
20.	1,3,3‐Trimethyl‐2‐hydroxymethyl‐3,3‐dimethyl‐4‐(3‐methylbut‐2‐enyl)‐cyclohexene	C_15_H_26_O	222	38.398	2.329104	550,198	Alkene	Antibacterial	Peng et al. (2017)

### 3.3 In Vitro Antioxidant Activity Study

#### 3.3.1 Determination of Total Phenolic Content

Following the Gallic acid calibration curve (*Y* = 5.877*x* + 0.0475; *R* ^2^ = 0.9936), the total amount of phenolic compounds in MOIB was 315.21 ± 0.39 mg GAE/g.

#### 3.3.2 Determination of Total Flavonoid Content

From the quercetin calibration curve (*Y* = 1.022*x* + 0.114; *R* ^2^ = 0.9866), the TFC was found to be 308.80 ± 1.49 mg QE/g dry extract in MOIB.

#### 3.3.3 DPPH Free Radical Scavenging Assay

**FIGURE 2:**
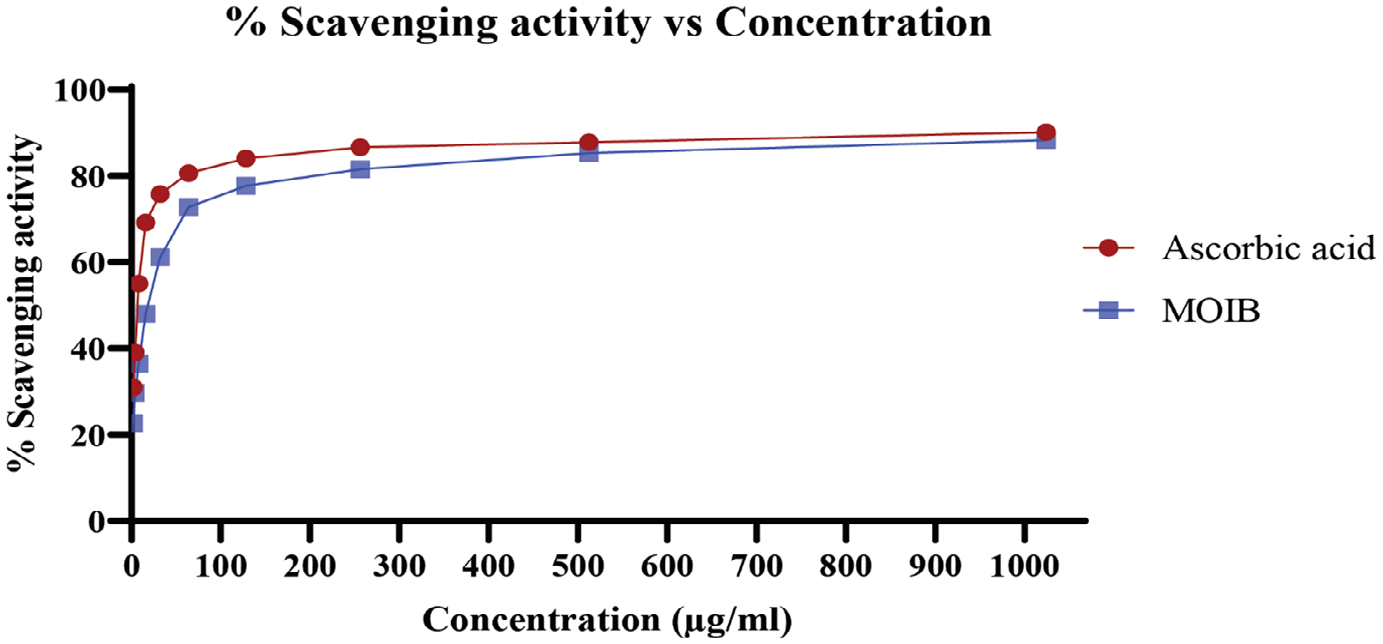
Illustration of DPPH free radical scavenging activity of standard (ascorbic acid) and MOIB.

In the DPPH free radical scavenging assay, both ascorbic acid and MOIB exhibited concentration‐dependent radical scavenging effects with IC_50_ values of 6.3848 and 19.6679 μg/mL (Figure 2).

#### 3.3.4 Reducing Power Assay

**FIGURE 3:**
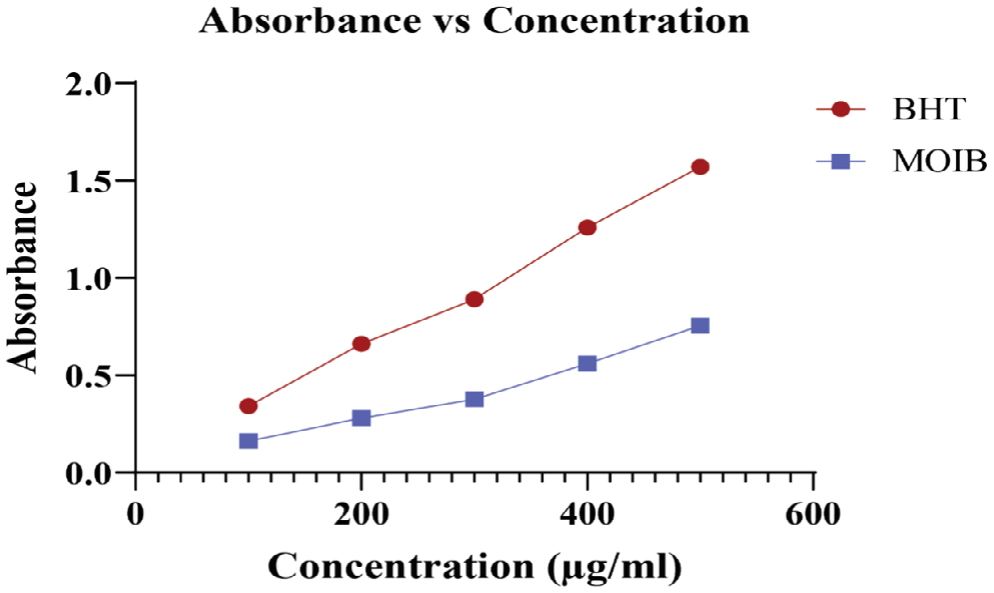
Illustration of reducing activity of MOIB compared to the reference standard (BHT).

In the reducing power assay, BHT and MOIB showed RC_50_ values of 1873.41 and 1752.76 μg/mL, respectively (Figure 3).

### 3.4 Acute Toxicity Studies

In an acute toxicity study, a single high dose of 5000 mg/kg of MOIB did not cause any deaths in terms of long‐term and short‐term observation periods. Additionally, the animals showed no signs of toxicity throughout the 14‐day study period.

### 3.5 Anxiolytic Activity Study

#### 3.5.1 Elevated Plus Maze Test

**FIGURE 4:**
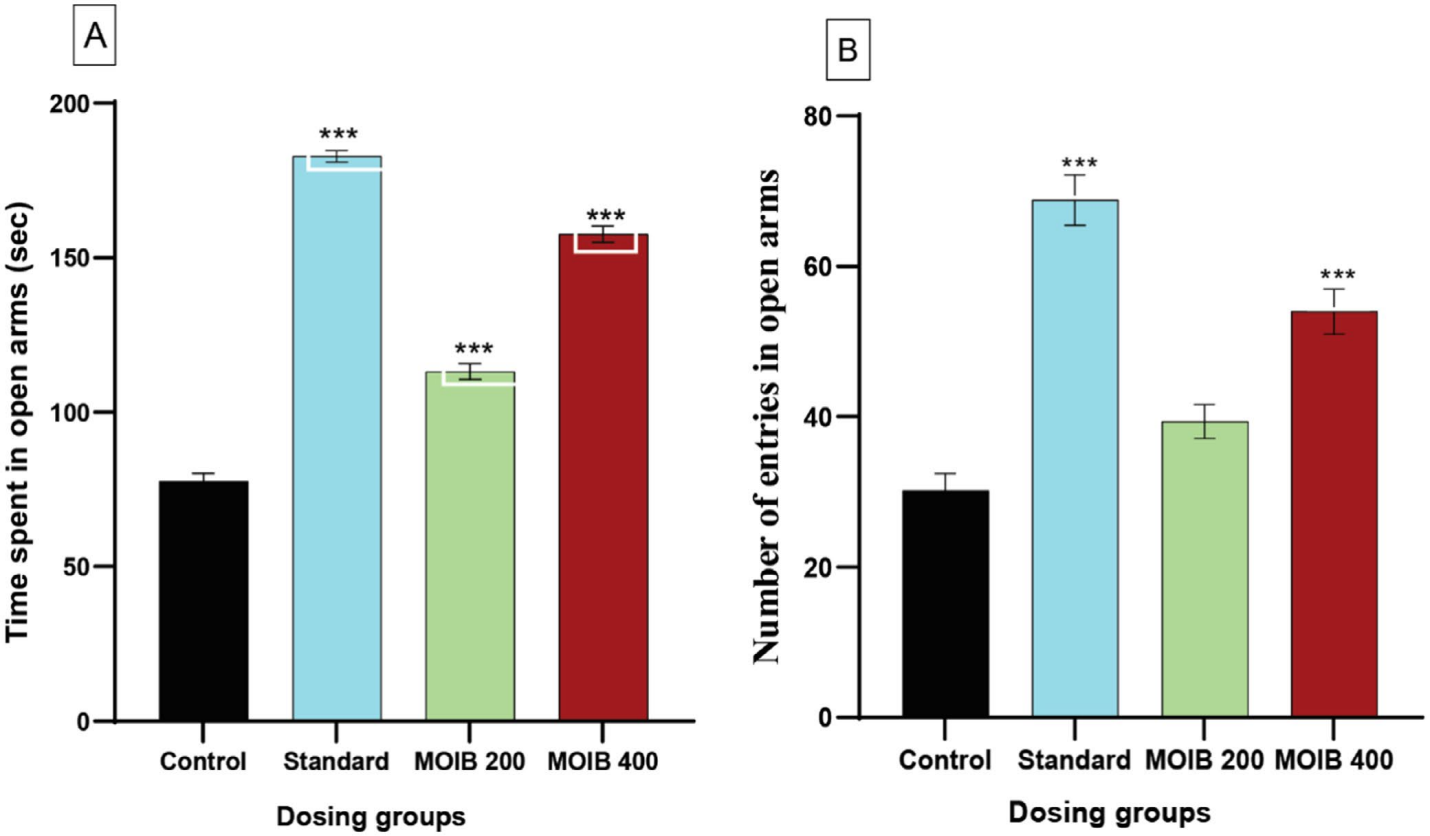
Effect of diazepam (standard) and MOIB in elevated plus‐maze test, (A) time spent in open arms; (B) number of entries into open arms; and *p < 0.5, **p < 0.01, and ***p < 0.001 were considered statistically significant.

In EPM, MOIB demonstrated a significant increase in the time spent and number of entries in open arms in a dose‐dependent manner in comparison to that of the control, indicating anxiolytic activity. Moreover, the standard showed maximum effect evidenced by time spent and number of entries in open arms (Figure 4).

#### 3.5.2 Hole Board Test

**FIGURE 5:**
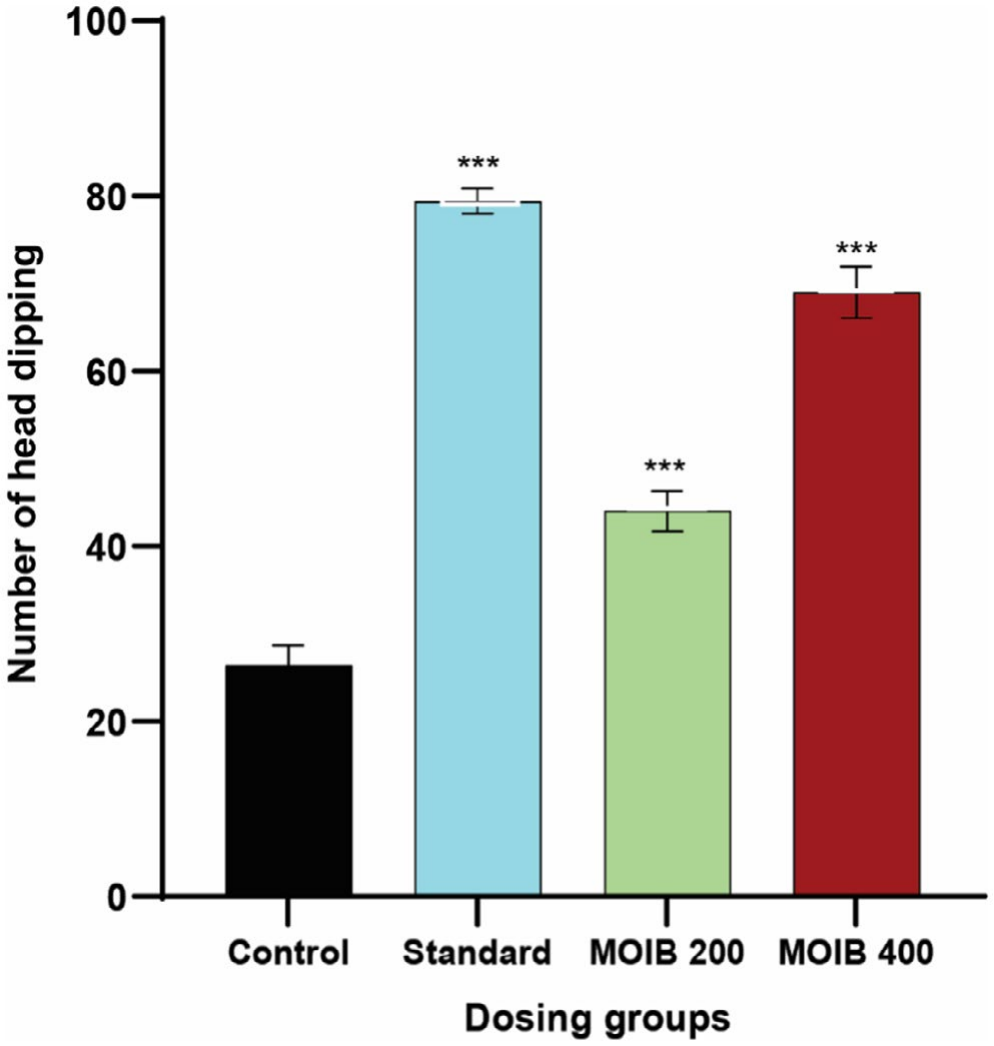
The effect of MOIB in the hole board test, where *p < 0.5, **p < 0.01, and ***p < 0.00 were regarded as statistically significant.

In HBT, a dose‐dependent increase in the number of head dipping was demonstrated by MOIB compared to that of the control, which suggests anxiolytic activity. However, the standard drug diazepam at 1 mg/kg exhibited the highest number of head dipping (Figure 5).

### 3.6 Locomotor Activity Study

#### 3.6.1 Open Field Test

**FIGURE 6:**
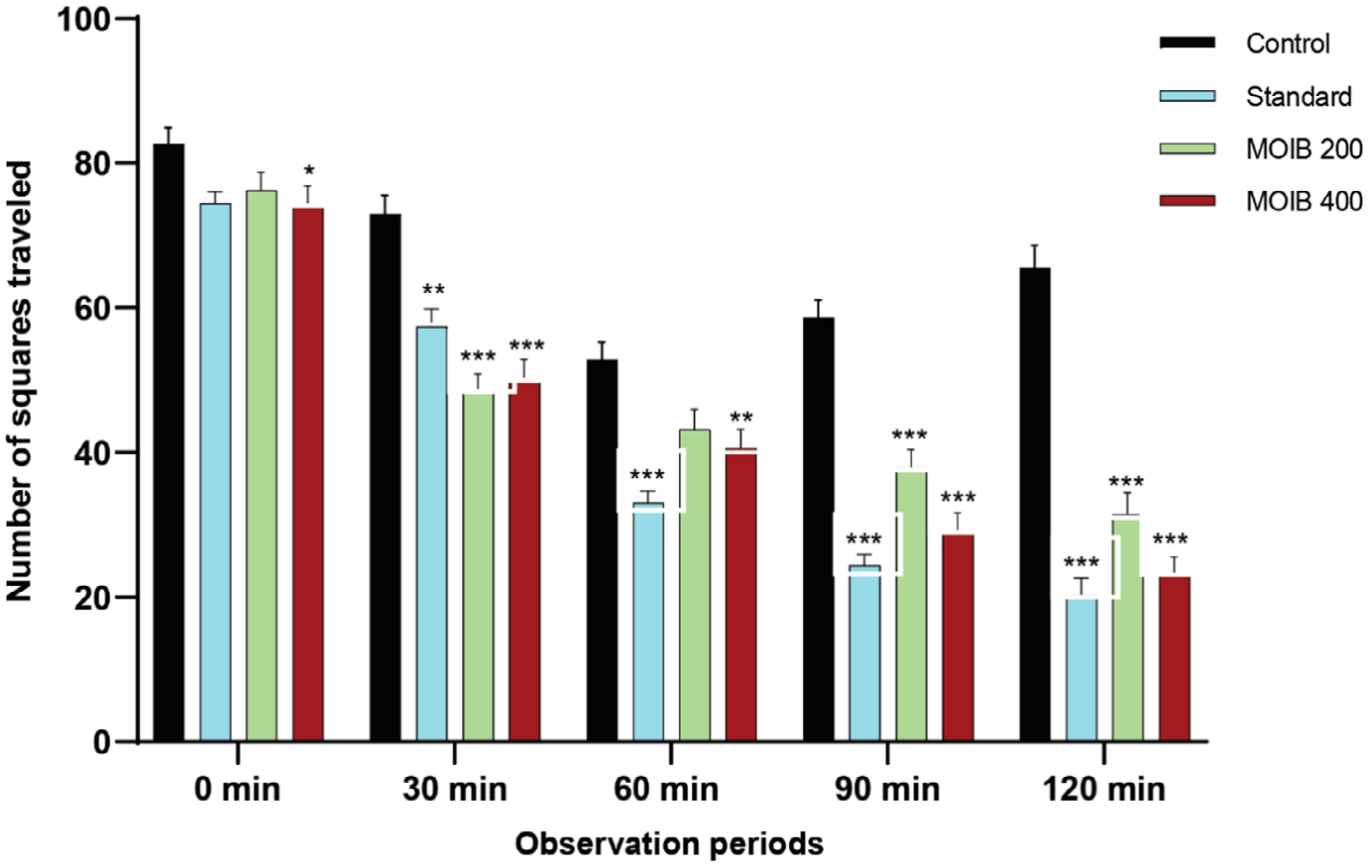
Effect of diazepam (standard) and MOIB in open field test; the number of squares traveled at the different intervals were 0 min, 30 min, 60 min, 90 min, and 120 min, and *p < 0.5, **p < 0.01, and ***p < 0.001 were reflected statistically significant.

In OFT, both the lower and higher doses of MOIB (200 and 400 mg/kg, respectively) significantly dwindled the number of movements from the 2^nd^ to 5^th^ observation periods when compared to that of the control, and it followed not only a dose‐dependent but also time‐dependent pattern (Figure 6).

#### 3.6.2 Hole Cross Test

**FIGURE 7:**
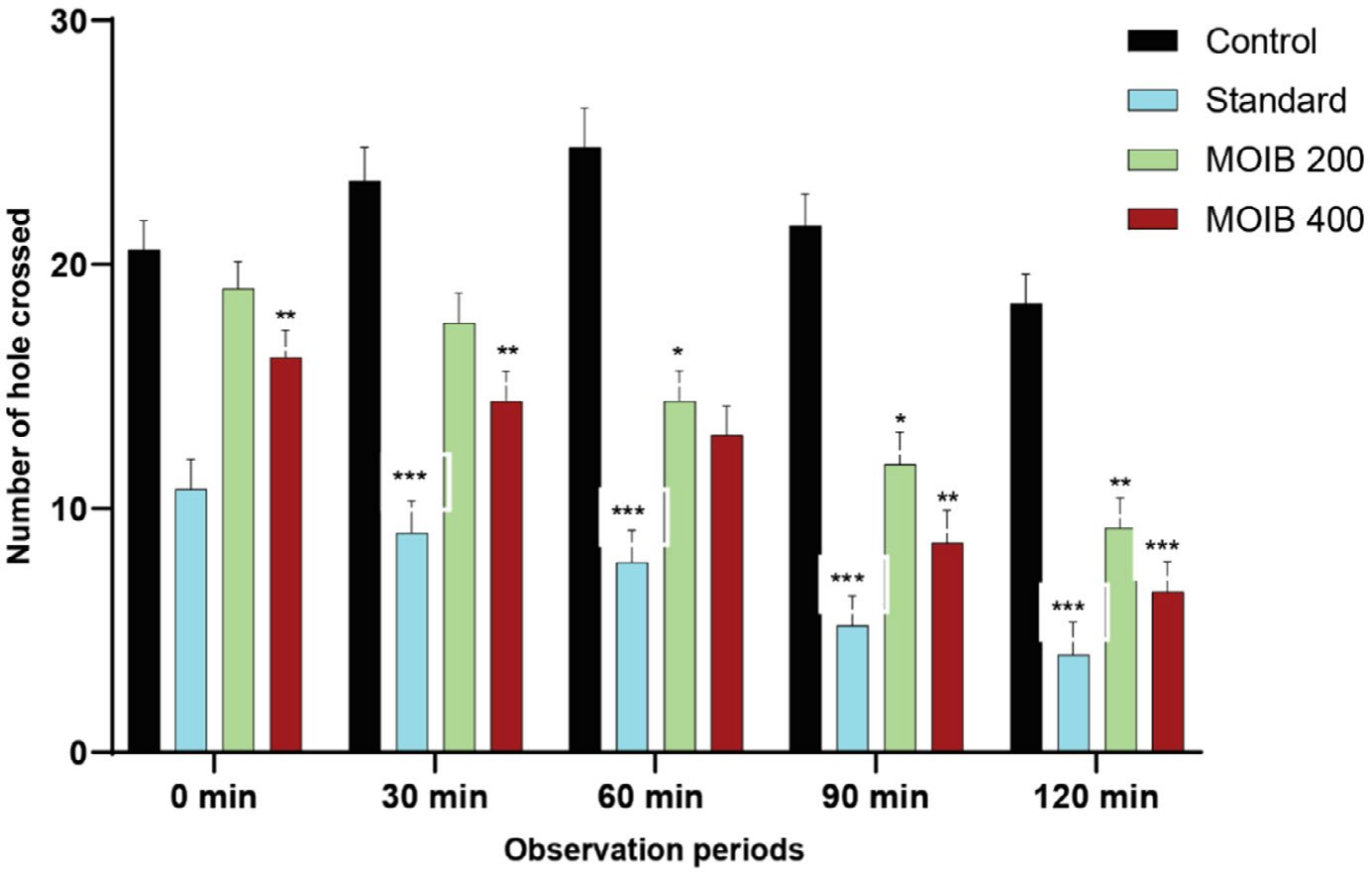
Effect of standard and MOIB in hole cross test; the number of holes crossed at the different time intervals were 0 min, 30 min, 60 min, 90 min, and 120 min where *p < 0.5, **p < 0.01, and *** *p* < 0.001 were examined statistically significant.

In HCT, the total number of holes crossed was significantly diminished by experimental mice after oral administration of MOIB (200 and 400 mg/kg), which was evident from the 2^nd^ to 5^th^ observation periods as compared to that of the control group. Moreover, mice treated with diazepam (1 mg/kg) also exhibited similar effects (Figure 7).

### 3.7 Antidepressant Activity Study

#### 3.7.1 Forced Swimming Test

**FIGURE 8:**
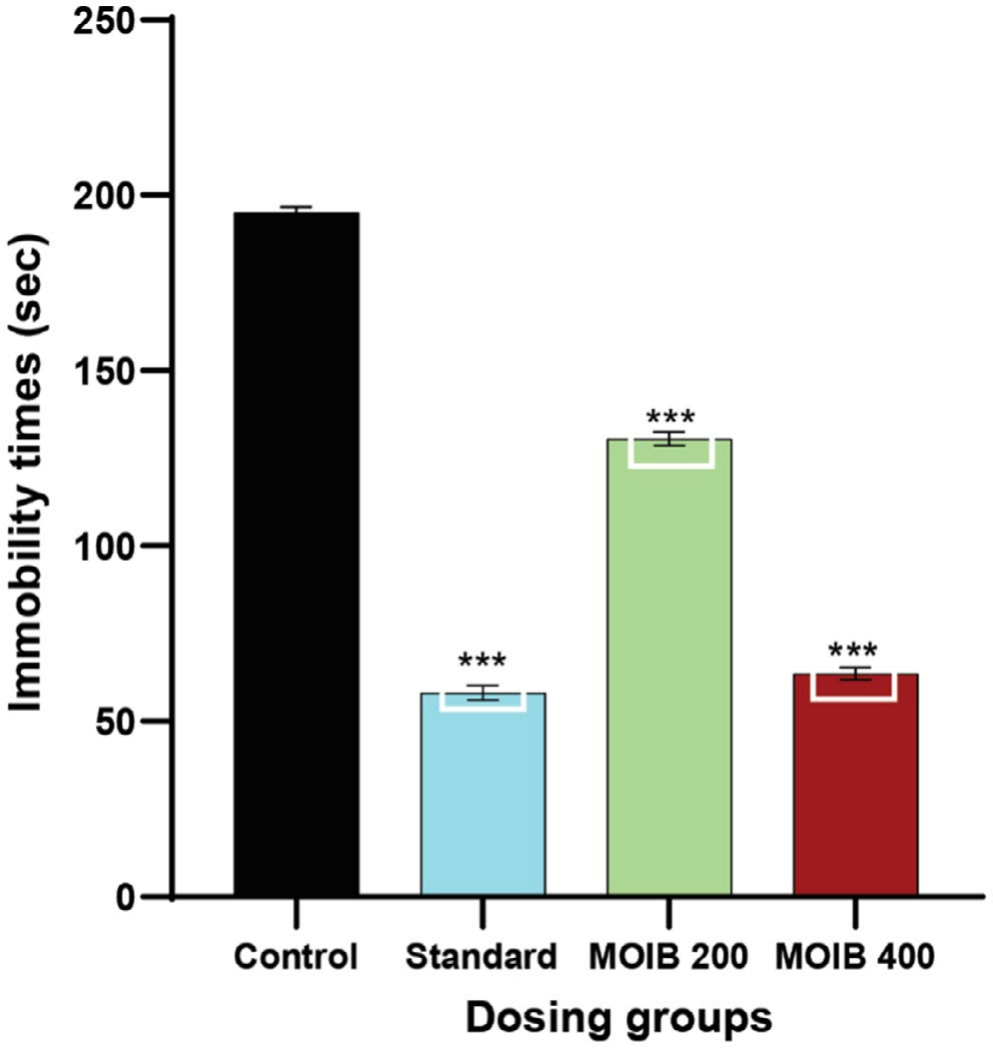
Effect of standard and MOIB in FST, where *p < 0.5, **p < 0.01, and *** < 0.001 were considered statistically significant.

In this study, MOIB at both doses (200 and 400 mg/kg) displayed notable results compared to the control group. It was found that MOIB showed a significant (***p < 0.001) reduction in immobility time, suggesting an anti‐depressant effect illustrated in Figure 8.

#### 3.7.2 Tail Suspension Test

**FIGURE 9:**
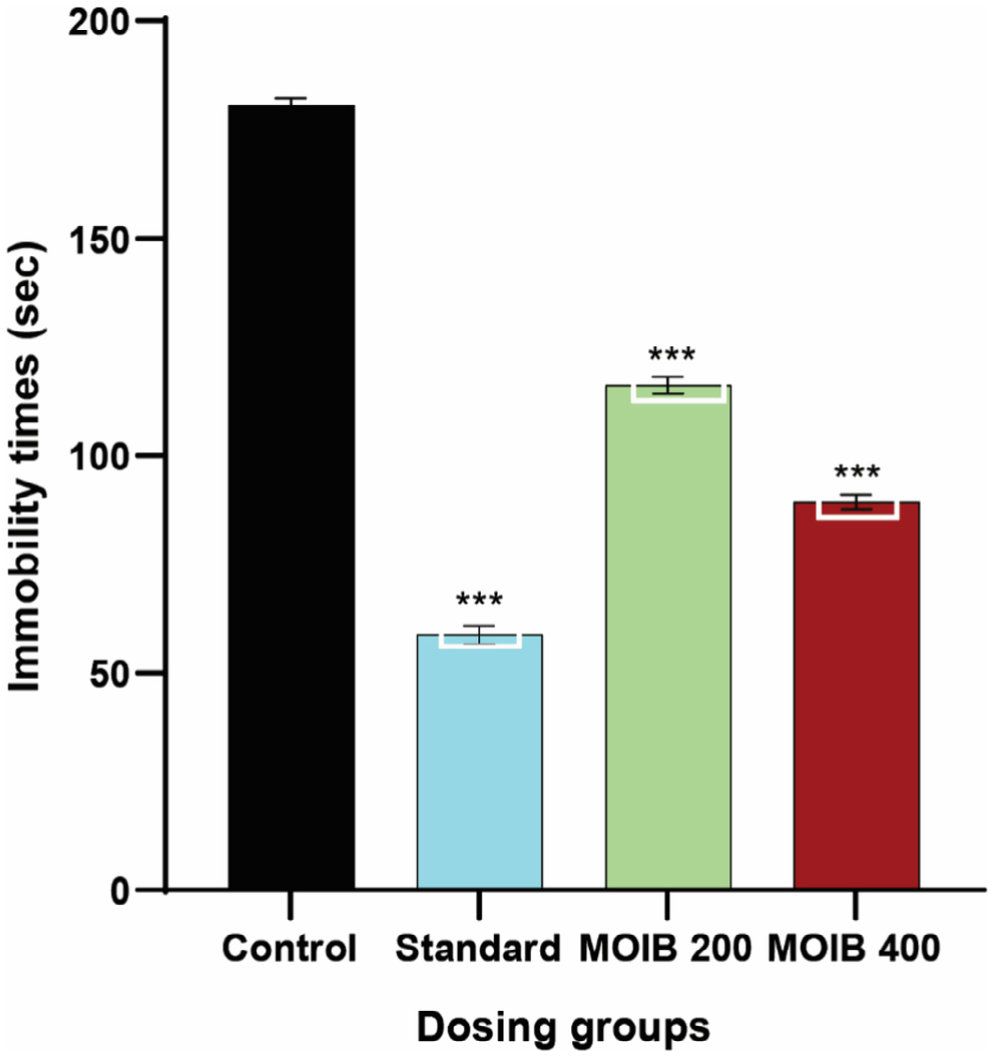
Effect of standard and MOIB in TST, where *p < 0.5, **p < 0.01, and *** *p* < 0.001 were considered statistically significant.

In the tail suspension test, similar anti‐depressant effects were observed by administered doses of MOIB (200 and 400 mg/kg) through the reduction of immobility time compared to the control group. In addition, the highest antidepressant activity was exhibited by the standard, as displayed in Figure 9.

### 3.8 In Silico Study

#### 3.8.1 ADME Analysis

ADME analysis revealed a satisfactory pharmacokinetics profile of the selected 7 compounds. Moreover, they followed Lipinski's Rules and Veber's Rules (Table 3). Again, the ADME analysis of all the compounds is given as Table S1.

**TABLE 3:** ADME profiling of the selected 7 compounds from MOIB.

Chemical identifier	Lipinski rules					Veber rules		Pharmacokinetics	
	MW (g/mol) < 500	HBA < 10	HBD < 5	Log P ≤ 5	Lipinski violation	nRB ≤ 10	TPSA ≤ 140	GI absorption	BBB permeability
CID 550198	222.37	1	1	4.09	No	3	20.23 Å^2^	High	Yes
CID 10393	138.16	2	2	0.93	No	2	40.46 Å^2^	High	Yes
CID 74465	192.25	2	0	2.79	No	3	26.30 Å^2^	High	Yes
CID 28554	284.78	3	1	2.18	No	9	41.57 Å^2^	High	Yes
CID 3042	309.49	3	0	4.40	No	8	29.54 Å^2^	High	Yes
CID 544080	186.33	1	1	3.75	No	8	20.23 Å^2^	High	Yes
CID 12729	212.33	2	0	3.86	No	11	26.30 Å^2^	High	Yes

Abbreviations: BBB, Blood–Brain Barrier; GIA, Gastrointestinal Absorption; HBA, Hydrogen Bond Acceptor; HBD, Hydrogen Bond Donor; Log P, Lipophilicity; MW, Molecular Weight; NRB, Number of Rotable Bonds; TPSA, Topological Polar Surface Area.

#### 3.8.2 Toxicity Analysis

None of the selected compounds demonstrated Ames toxicity, hepatotoxicity, or mutagenicity. Besides, those compounds also exhibited other satisfactory toxicity profiles (Table 4).

**TABLE 4:** Toxicity profiling of the selected seven compounds.

Chemical identifier	Types of toxicity					
	Ames Toxicity	Hepatotoxicity (probability)	Immunogenicity (probability)	Carcinogenicity (probability)	Mutagenicity (probability)	Predicted LD_50_ (mg/kg)
CID 550198	NAT	Inactive (0.81)	Inactive (0.91)	Inactive (0.68)	Inactive (0.63)	2100 mg/kg
CID 10393	NAT	Inactive (0.89)	Inactive (0.75)	Inactive (0.99)	Inactive (0.93)	
CID 74465	NAT	Inactive (0.71)	Inactive (0.99)	active (0.54)	Inactive (0.85)	1030 mg/kg
CID 28554	NAT	Inactive (0.91)	Active (0.70)	inactive (0.54)	Inactive (0.73)	
CID 3042	NAT	Inactive (0.99)	Active (0.92)	Active (0.5)	Inactive (0.79)	3730 mg/kg
CID 544080	NAT	Inactive (0.72)	Inactive (0.95)	Inactive (0.69)	Inactive (0.65)	
CID 550198	NAT	Inactive (0.81)	Inactive (0.91)	Inactive (0.68)	Inactive (0.63)	1 mg/kg

Abbreviation: NAT, Non‐Ames toxic.

#### 3.8.3 Molecular Docking Analysis

Molecular docking is used to find the most effective intermolecular interaction between a protein (macromolecule) and a ligand (Islam, Aktaruzzaman, Islam, et al. 2025; Saif et al. 2025; Hasan et al. 2025). In our study, molecular docking was conducted on 7 selected compounds based on the ADME and toxicity profiling, along with the reference drugs to compare the findings. It was found that two compounds with CID 550198 and CID 10393 showed good binding affinity to the targeted three receptors. For potassium channel receptors, CID 550198 and CID 10393 displayed the highest binding affinity of −5.62 and −5.034 kcal/mol, compared to that of standard diazepam (−5.096 kcal/mol). Again, the remarkable binding affinity of −5.086 and −5.429 kcal/mol was revealed by CID 550198 and CID 10393 to the human gamma‐aminobutyric acid GABA_A_ receptor whereas diazepam showed a binding affinity of −5.42 kcal/mol. Furthermore, while standard imipramine hydrochloride exhibited a binding affinity of −7.587 kcal/mol to the human serotonin transporter, CID 550198 and CID 10393 showed a binding affinity of −6.938 and −6.826 kcal/mol to that targeted receptor. All the docking scores are presented in Table 5.

**TABLE 5:** The binding affinity of the selected ligands to the targeted proteins.

Sl. no.	Chemical identifier	Compound name	Docking score (Kcal/mol)		
			4UUJ	6X3X	5I6X
1.	CID 550198	1,3,3‐Trimethyl‐2‐hydroxymethyl‐3,3‐dimethyl‐4‐(3‐methylbut‐2‐enyl)‐cyclohexene	−5.62	−5.086	−6.938
2.	CID 10393	Benzeneethanol, 4‐hydroxy—	−5.034	−5.429	−6.826
3.	CID 74465	Ethyl 2,4,6‐trimethylbenzoate	−4.189	−3.76	−5.523
4.	CID 28554	Clofexamide	−3.302	−2.699	−4.575
5.	CID 3042	Dicyclomine	−4.129	−2.785	−4.358
6.	CID 544080	2‐Methyl‐5‐undecanol	−0.6	−0.616	−0.905
7.	CID 12729	Ethyl 10‐undecenoate	1.245	NA	1.174
8.	CID 3016	Diazepam	−5.096	−5.42	NA
9.	CID 3696	Imipramine hydrochloride	NA	NA	−7.587

#### 3.8.4 Interpretation of Protein‐Ligand Interactions

**FIGURE 10:**
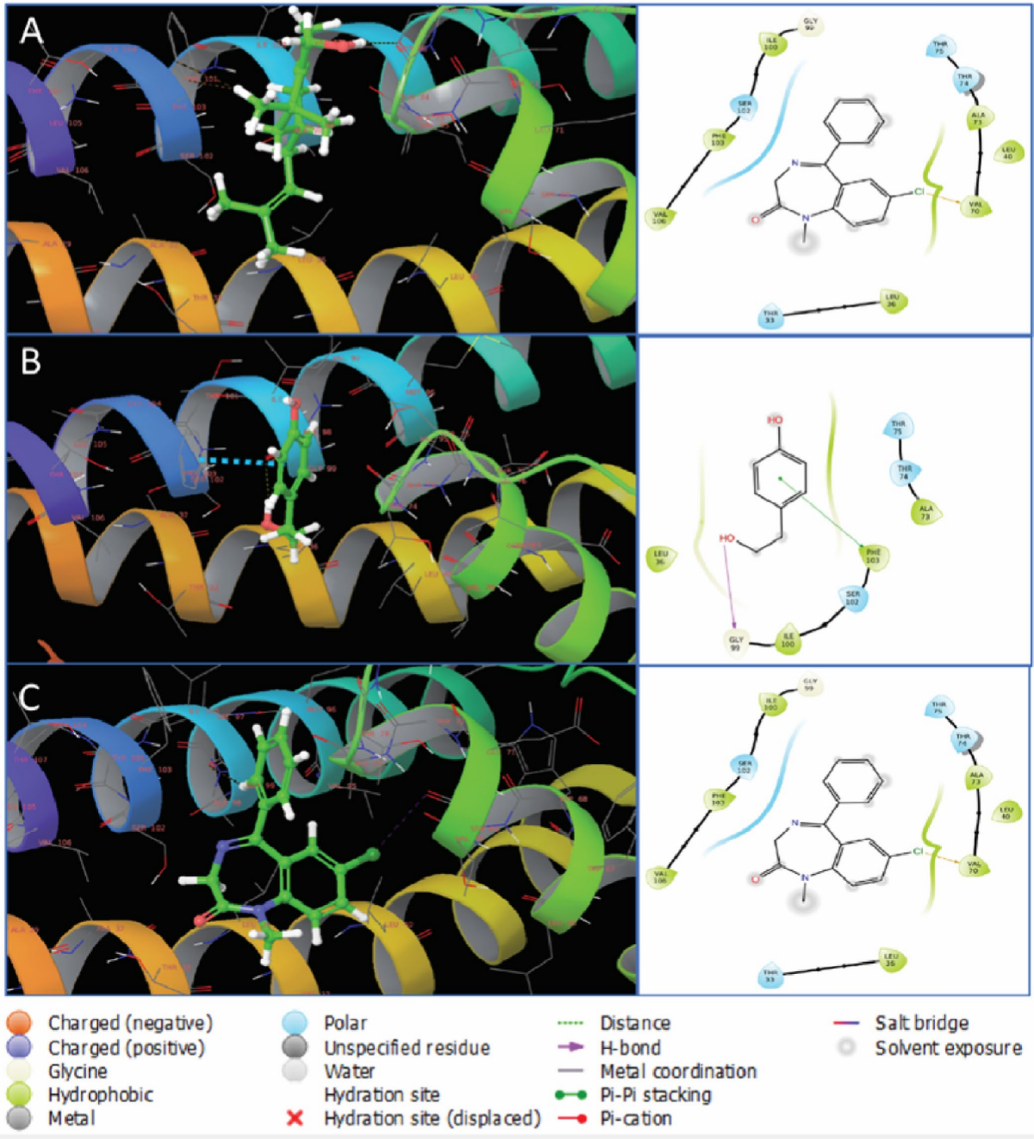
Binding of CID 550198, CID 10393, and CID 3016 (diazepam) with (4UUJ). The left side of the figure shows the 3D structure, while the right side shows the 2D structure of the protein‐ligand complex. In the 3D structure, ligand molecules are represented with green color. Here, (A–C) denote CID 550198, CID 10393, and CID 3016, respectively.

The interactions between CID 550198 and CID 10393 and the targeted three receptors revealed several hydrogen bonds, polar bonds, and hydrophobic bonds shown in Table 6. The interactions indicate that CID 550198 bound to potassium channel receptor (4UUJ) through the formation of four polar bonds with SER102, THR75, THR74, THR33 and eight other bonds with SER102, THR75, THR74, THR33 whereas CID 10393 interacted through one hydrogen bond with GLY99, three polar bonds with THR75, THR74, SER102 and four other bonds with ALA73, PHE103, ILE100, LEU36. In contrast, diazepam (CID 3016) interacted with 4UUJ by forming three polar bonds with SER102, THR75, ALA73 and nine other bonds with VAL106, PHE103, ILE100, GLY99, LEU36, ALA73, LEU40, VAL70, THR33 illustrated in Figure 10.

**TABLE 6:** Molecular interaction of the selected ligands with targeted receptors.

Protein PDB ID	PubChem ID	H‐bond	Polar bond	Other bonds
4UUJ	CID 550198		**SER102, THR75**, THR74, **THR33**	**VAL106, PHE103, ILE100, GLY99 ALA73, LEU40, VAL70, LEU36**
	CID 10393	**GLY99**	**THR75**, THR74, **SER102**	**ALA73**, **PHE103**, **ILE100**, **LEU36**
	CID 3016		SER102, THR75, ALA73	VAL106, PHE103, ILE100, GLY99, LEU36, ALA73, LEU40, VAL70, THR33
6X3X	CID 550198		HIS142, SER195	ARG194, TRP196, **ARG197**, TYR199, GLU138, CYS139, PRO140, MET58, MET141, LEU143, GLU144, ARG232, TYR282
	CID 10393	SER195	HIS142	ARG194, TRP196, **ARG197**, TYR199, MET58, GLU138, PRO140, MET141, GLU144, TYR282, VAL280
	CID 3016	ARG197	SER61, THR73, HIS122, THR146	LYS105, LYS106, ARG144, LYS118, ASP146, ASP148, ALA119, HIS122, ASP75, ASP57S, PRO64
5I6X	CID 550198	**PHE335**	**SER336**, ASN177, **SER438**, **SER439**	ASP98, **ALA96**, **TYR95**, **GLY338**, **PHE341**, **VAL501**, **VAL343**, ALA169, ILE172, ALA173, **TYR176**, **GLY442**, LEU443
	CID 10393	**SER439**	**SER438**, ASN177	ASP98, **TYR95**, **PHE341**, **VAL343**, ALA169, ILE172, ALA173, **TYR176**, LEU443, **GLY442**, ILE266
	CID 3696	ASN98	SER336, THR497, ASN368, ASN101, SER439, SER438	ALA96, TRY95, VAL343, PHE341, GLY338, LEU337, PHE335, PHE334, VAL501, GLY498, GLU439, ILE175, TYR175, TRY176, GLY442

*Note:* Bold indicates the common interacting residues found in both selected compounds and standard drugs.

**FIGURE 11:**
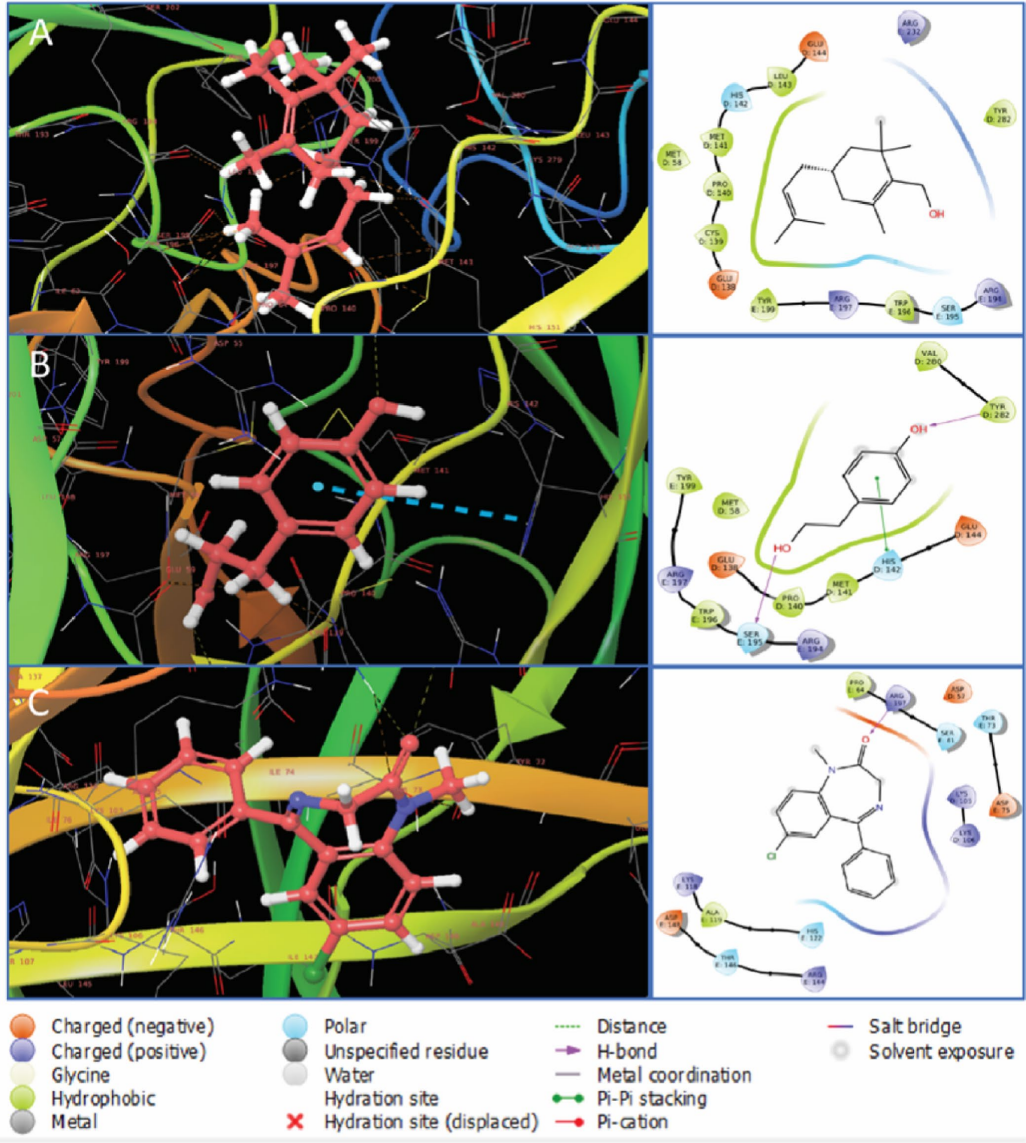
Binding of CID 550198, CID 10393, and CID 3016 (diazepam) with (6X3X). This protein‐ligand complex has a 3D structure on the left and a 2D structure on the right. In the 3D structure, ligand molecules are represented with maroon color. Here, A, B, and C indicate CID 550198, CID 10393, and CID 3016, respectively.

When it comes to molecular interactions of the selected two compounds with human gamma‐aminobutyric acid GABA_A_ (6X3X), it was found that CID 550198 formed two polar bonds with HIS142, SER195 as well as thirteen other bonds with ARG194, TRP196, ARG197, TYR199, GLU138, CYS139, PRO140, MET58, MET141, LEU143, GLU144, ARG232, TYR282 of GABAA; in contrast, CID 10393 interacted through one hydrogen bond with SER195, one polar bond with HIS142, and eleven other bonds with ARG194, TRP196, ARG197, TYR199, MET58, GLU138, PRO140, MET141, GLU144, TYR282, VAL280. Moreover, one hydrogen bond with ARG197, four polar bonds with SER61, THR73, HIS122, THR146, and eleven other bonds with LYS105, LYS106, ARG144, LYS118, ASP146, ASP148, ALA119, HIS122, ASP75, ASP57S, PRO64 were formed by diazepam (CID 3016) shown in Figure 11.

**FIGURE 12:**
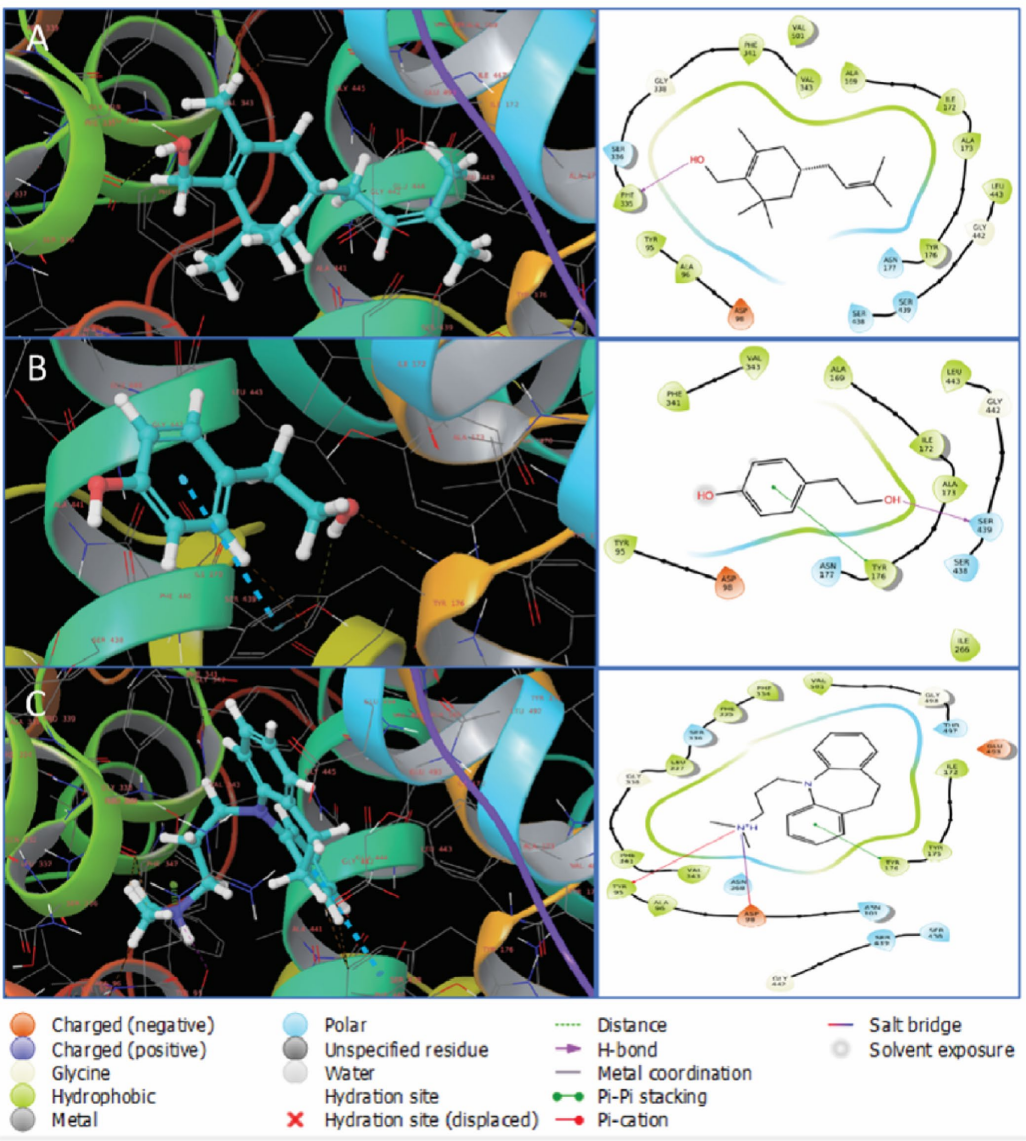
Binding of CID 550198, CID 10393 and CID 3696 (Imipramine) with (5I6X). In the protein‐ligand complex, there is a 2D structure on the right side and a 3D structure on the left. In the 3D structure, ligand molecules are represented with cyan color. Here, (A–C) denote CID 550198, CID 10393, and CID 3696, respectively.

Furthermore, interaction analysis of the selected two compounds with human serotonin transporter (5I6X) indicates that CID 550198 interacted with 5I6X forming one hydrogen bond with PHE335, four polar bonds with SER336, ASN177, SER438, SER439 and thirteen other bonds with ASP98, ALA96, TYR95, GLY338, PHE341, VAL501, VAL343, ALA169, ILE172, ALA173, TYR176, GLY442, LEU443; conversely, CID 10393 made one hydrogen bond with SER439, two polar bonds with SER438, ASN177, and eleven other bonds with ASP98, TYR95, PHE341, VAL343, ALA169, ILE172, ALA173, TYR176, LEU443, GLY442, and ILE266. In addition, imipramine (CID 3696) interacted with 5I6X by forming one hydrogen bond with ASN98, six polar bonds with SER336, THR497, ASN368, ASN101, SER439, and SER438 and fifteen other bonds with ALA96, TYR95, VAL343, PHE341, GLY338, LEU337, PHE335, PHE334, VAL501, GLY498, GLU439, ILE175, TYR175, TYR176, and GLY442, depicted in Figure 12.

#### 3.8.5 Molecular Dynamics Simulation

Molecular dynamics (MD) simulation plays a crucial role in post‐dock analysis, enabling the exploration of time‐dependent stability and atom movements within the biological environment (Aktaruzzaman et al. 2025; Talukder et al. 2025). Essential analysis within MD simulations includes RMSD, RMSF, SASA, and RG, collectively offering a comprehensive grasp of the molecular behavior of protein‐ligand complexes. These analyses were conducted following a 100 ns dynamics trajectory for the three apoproteins (4UUJ, 6X3X, and 5I6X) and their corresponding complexes.

##### 3.8.5.1 RMSD Analysis

**FIGURE 13:**
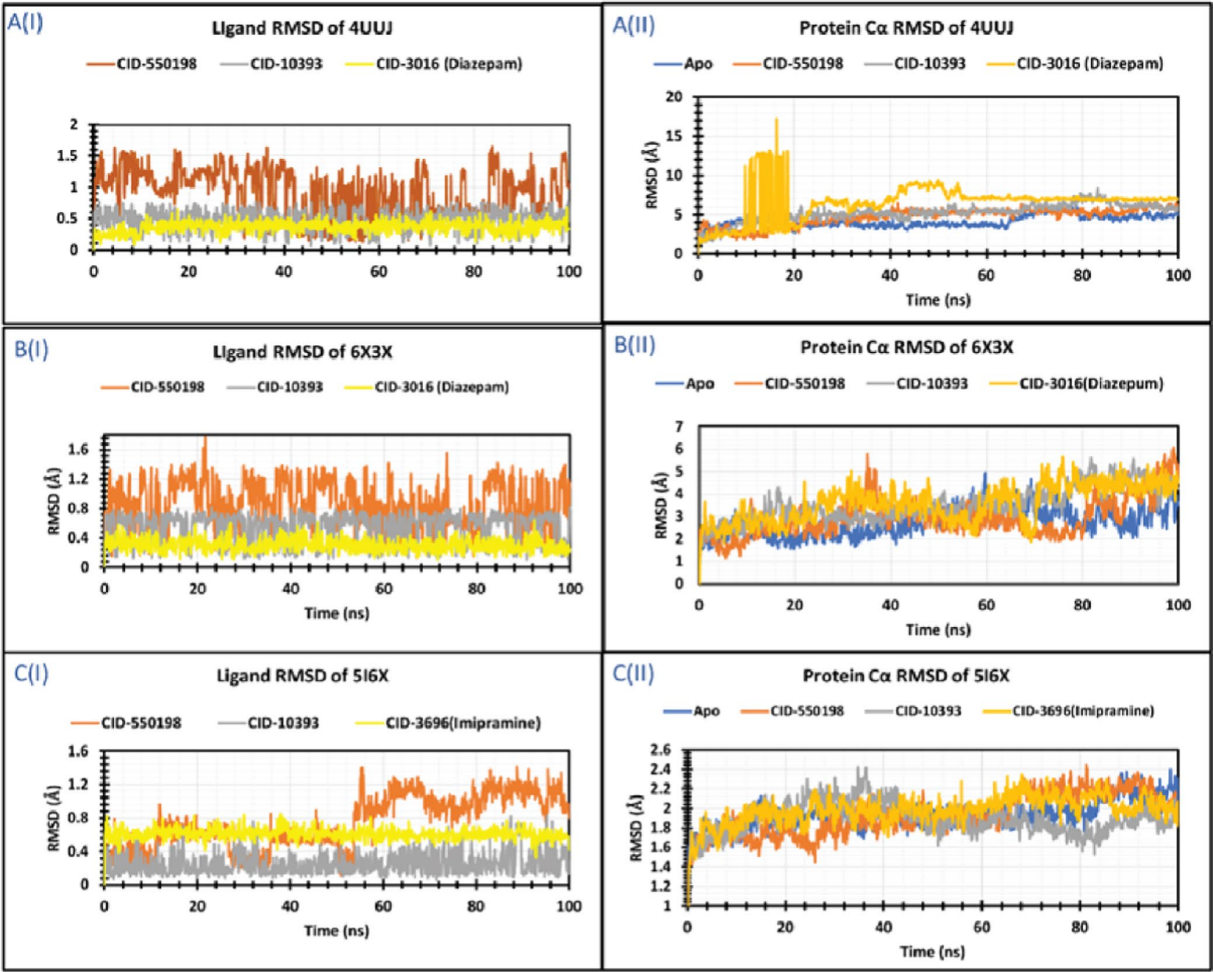
RMSD values of 4UUJ, 6X3X, and 5I6X in complex with the selected three compounds extracted from Cα atoms of the complex system.

For 4UUJ, 4UUJ‐CID550198, 4UUJ‐CID10393, and 4UUJ‐CID3016 (Diazepam), the average Cα‐RMSD values were 4.13, 4.76, 5.26, and 6.54 Å, respectively. The initial three simulated complexes exhibited consistently low trajectories throughout the simulation runtime. Conversely, the control complex involving Diazepam displayed high fluctuations from 10 ns to 18 ns, peaking at 16.84 Å, followed by moderate fluctuations from 40 ns to 55 ns. Ultimately, all complexes reached an equilibrium state over the period. Next, the average Cα‐RMSD values were found to be 2.74, 2.98, 3.51, and 3.59 Å for 6X3X, 6X3X‐CID550198, 6X3X‐CID10393, and 6X3X‐CID3016 (Diazepam), respectively. Besides, 6X3X‐CID550198 and 6X3X‐CID3016 complexes showed relatively moderate fluctuations after 80 ns and 70 ns, respectively. The other two complexes demonstrated comparatively mild fluctuations throughout the MD simulation. Then, in the case of 5I6X, 5I6X‐CID550198, 5I6X‐CID10393, and 5I6X‐CID3696 (Imipramine), the average Cα‐RMSD values were 1.93, 1.93, 1.89, and 1.98 Å (Figure 13).

##### 3.8.5.2 RMSF Analysis

**FIGURE 14:**
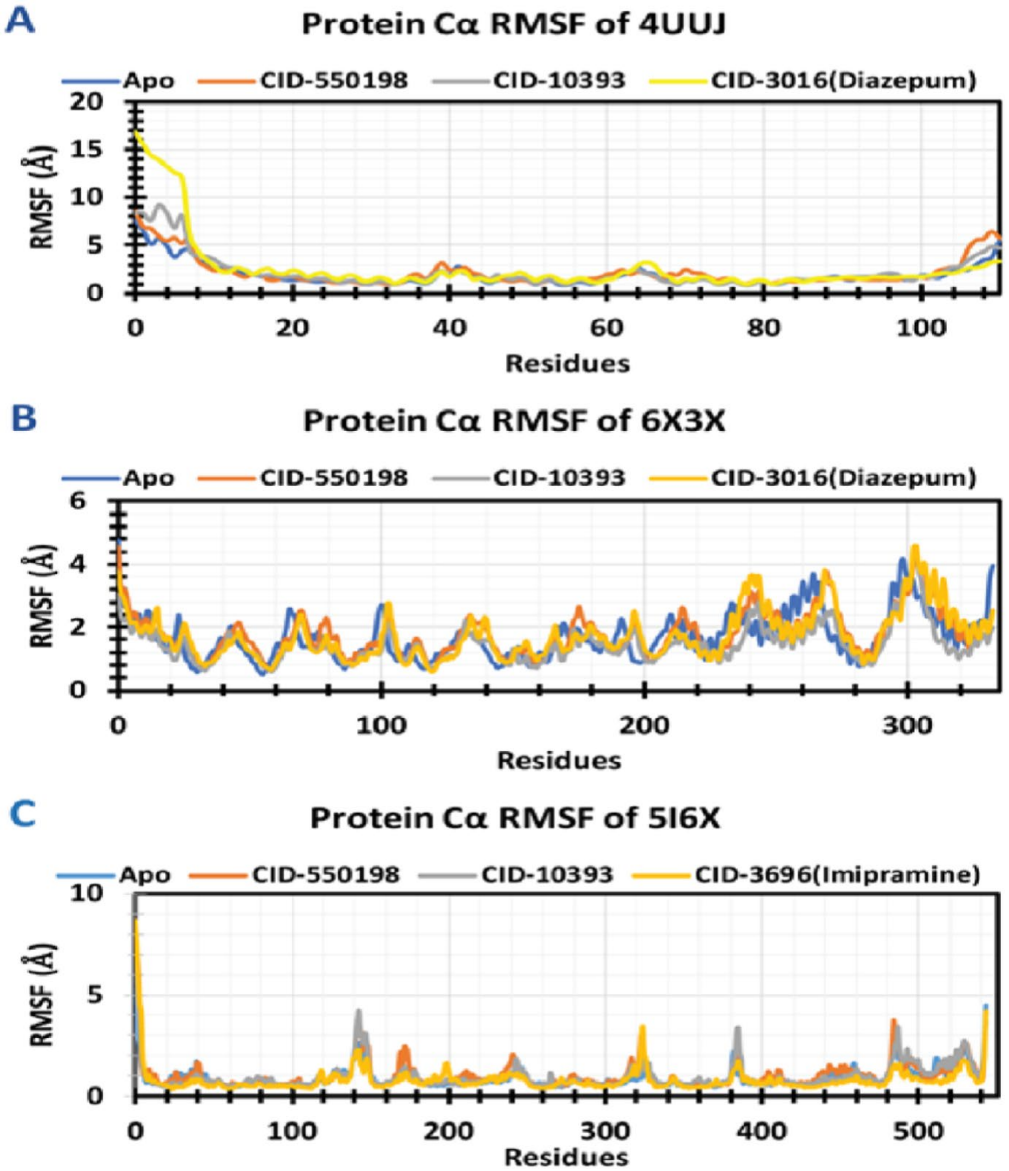
RMSF values extracted from protein‐ligand complexes. Here, the RMSF of Apo, CID 550198, and CID 10393 are represented by blue, orange, and gray colors, respectively, whereas the RMSF of CID 3016 (diazepam) and CID 3696 (imipramine) is represented by yellow color.

Notably, none of these complexes exhibited significant fluctuations during the simulation, maintaining an equilibrium state throughout. The apo 4UUJ, along with its three complexes, exhibited similar RMSF values across the amino acid residues between 1 and 10. However, the diazepam complex displayed significant fluctuations within this region compared to the other complexes. Besides, comparatively low RMSF values were demonstrated by all the complexes throughout the remaining amino acid residues of the targeted protein. Similarly, in the case of 6X3X, the apoprotein and its associated complexes showed significant RMSF values in different segments of amino acid residues, including 237–244, 268–271, and 299–314. On the other hand, 5I6X and its complexes displayed minimal fluctuations across all residues of amino acid (Figure 14).

##### 3.8.5.3 Radius of Gyration (RG) and Solvent Accessible Surface Area (SASA)

**FIGURE 15:**
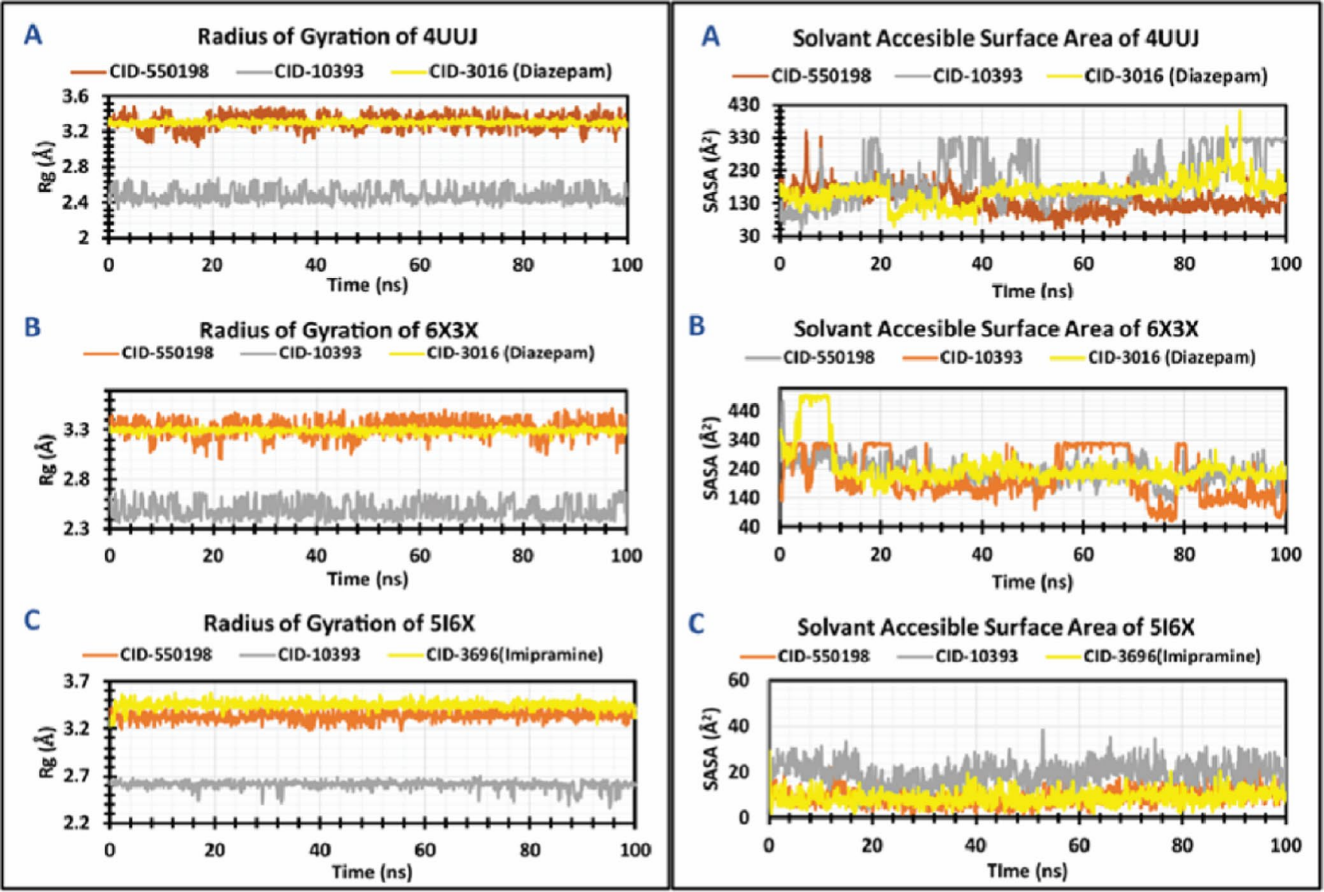
The radius of gyration (RG) and solvent‐accessible surface area (SASA) of the protein‐ligand complex were calculated from the 100 ns simulation. The RG and SASA values of the selected compounds CID 550198, CID 10393, and control (CID 3016 and CID 3696) in complex with the targeted proteins are represented by orange, gray, and yellow color, respectively.

The 4UUJ complexes with CID 550198, CID 10393, and CID 3016 displayed an average RG value of 2.5, 3.3, and 3.2 Å, respectively, while these values were 2.5, 3.2, and 3.3 Å for 6X3X complexes. Again, the average RG values of 2.6, 3.4, and 3.4 Å were found to be exhibited by 5I6X complexes. Besides, all the protein complexes displayed a stable trajectory without significant variations throughout the simulation period. On the other hand, when it comes to SASA, the 4UUJ‐CID550198 complex demonstrated a relatively consistent SASA trajectory characterized by minor fluctuations; in contrast, the 4UUJ‐CID10393 complex exhibited remarkable fluctuations at the time intervals of 15 ns to 20 ns, 33 ns to 51 ns, and subsequently after 74 ns. Similar fluctuation patterns were observed in terms of the 4UUJ‐CID 3016 complex, specifically within the period of 85 ns to 90 ns. Furthermore, in the case of 6X3X protein, the complex with CID 3016 initially displayed evident fluctuations from 3 ns to 12 ns, followed by a more stable trajectory. Moreover, an equilibrium behavior was observed for the 6X3X‐CID550198 complex, whereas the 4UUJ‐CID10393 complex revealed multiple instances of moderate to high fluctuations throughout the simulation time. Lastly, all 5I6X complexes maintained steady SASA trajectories, devoid of significant fluctuations (Figure 15).

#### 3.8.6 Principal Component Analysis (PCA)

**FIGURE 16:**
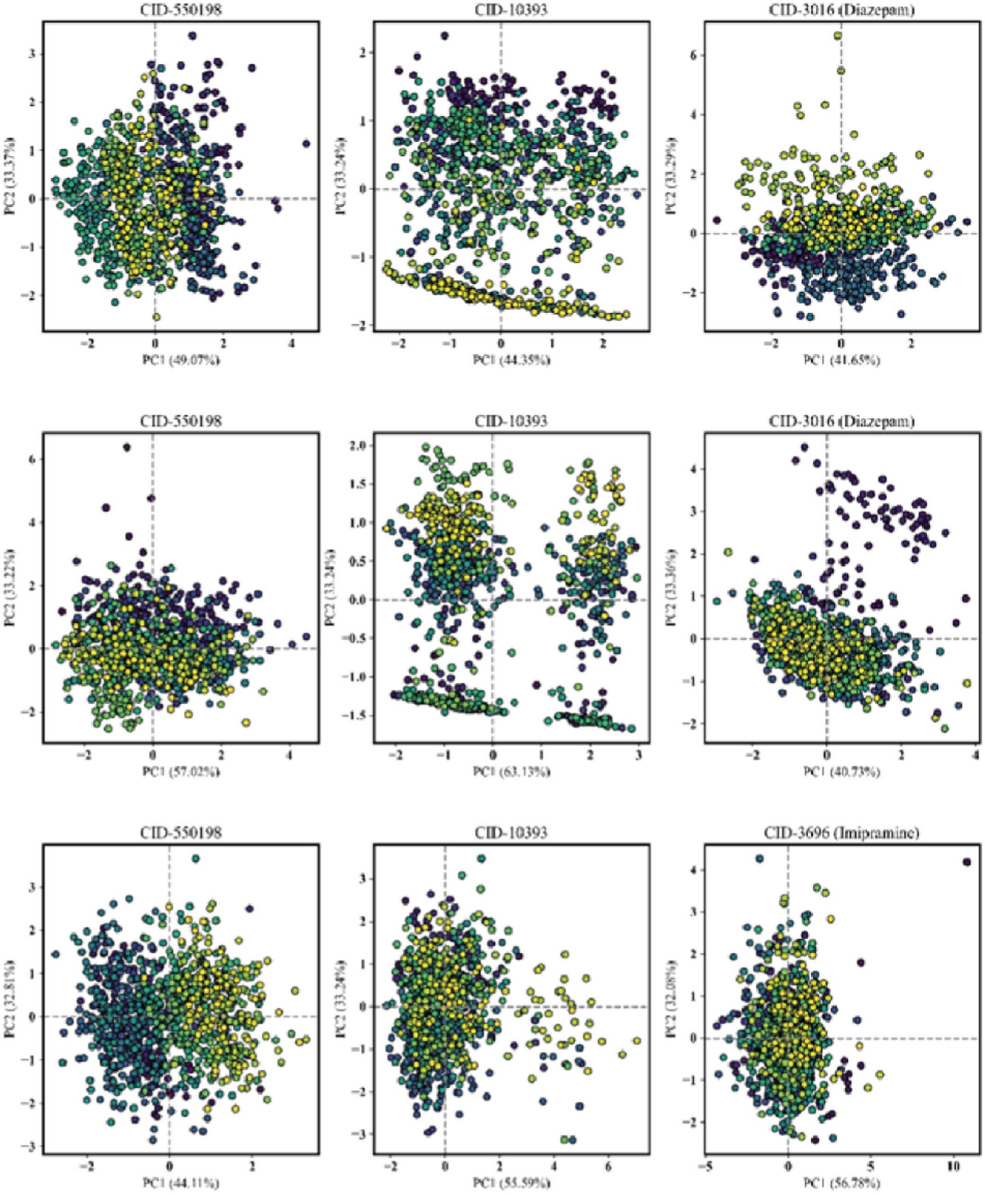
An analysis of principal components (PCA). Using a 100 ns simulation runtime trajectory, PCA was used to analyze the structural conformations of protein‐ligand complexes. Using Python v.3.10, the scikit‐learn library was used to carry out the analysis.

PCA demonstrates the distribution of variance along the principal components, shedding light on the significant modes of motion within each complex. In this analysis, for the 4UUJ‐ CID550198 complex, PC1 accounted for 49.07% of the total variance, while PC2 contributed 33.37%. Similarly, in the case of the 4UUJ‐CID10393 complex, PC1 represented 44.35% of the variance, with PC2 explaining 33.24%. Again, for the 4UUJ‐CID3016 complex, PC1 contributed to 41.65% of the variance, and PC2 explained 33.29%. On the other hand, the 6X3X‐CID550198 complex showcased PC1 representing 57.02% of the total variance, accompanied by PC2 accounting for 33.22%. Similarly, for the 6X3X‐CID10393 complex, PC1 contributed to 63.13% of the variance, while PC2 explained 33.24%. In the case of the 6X3X‐CID3016 complex, PC1 contributed to 40.73% of the variance, along with PC2 explaining 33.36%. At last, in terms of the 5I6X‐CID550198 complex, PC1 elucidated 44.11% of the overall variance, while PC2 accounted for 32.81%. Similarly, PC1 captured 55.59% of the variance for the 5I6X‐CID10393 complex, with PC2 accounting for 33.24%. Next, the 5I6X‐CID3696 complex exhibited PC1 contributing to 56.78% of the variance, and PC2 clarifying 32.08% (Figure 16).

## 4 Discussion

Historically, plants have been considered a principal source of valuable therapeutic agents for the treatment of various diseases (Koehn and Carter 2005). Plants are a superb source of biomolecules that can offer new approaches to treating many chronic illnesses, even in our modern age (Minogue 2018; Hasan et al. 2024). Clinically available antidepressants and anxiolytics also work through different mechanisms, including dopaminergic, noradrenergic, and serotonergic pathways (Berton and Nestler 2006). The drawbacks of these drugs include insufficient efficacy over the long term and unexpected side effects (Chen et al. 2015). In recent years, many researchers have explored alternative sources of drugs that appear to have fewer or no side effects (Ngan and Conduit 2011). A number of herbal plants have been studied for their antioxidant properties, including *Diospyros abyssinica*, *Pistacia lentiscus* , *Geranium sanguineum* (Krishnaiah et al. 2011); and *Musa sapientum, Capparis thonningii* for their antidepressant and anxiolytic properties (Ishola et al. 2015; Salako et al. 2019; Uddin et al. 2024). It is essential to develop animal models to develop herbal drugs. Experimental protocols based on validated animal studies are therefore essential for making relevant preclinical and clinical conclusions. In this study, we have investigated the possible antioxidant, anxiolytic, and antidepressant effects of MOIB using in vitro methods and in vivo models, along with in silico approaches.

Phytochemical screening of MOIB revealed the presence of alkaloids, flavonoids, phenols, steroids, glycosides, and terpenoids, consistent with previous studies on *Hygrophila spinosa* reporting similar secondary metabolites with diverse pharmacological properties (Agidew 2022; Uddin et al. 2024). Furthermore, the experimental samples showed no mortality, abnormal behavior, or neurological changes at the single high dose of 5000 mg/kg in the acute toxicity study, which indicates that doses of the crude extract used in the experiment were safe and effective.

Moreover, elevated levels of reactive oxygen species (ROS) in the brain resulting in oxidative stress (OS) may further cause neuroinflammation at both physiological and pathological levels (Salim 2014; Ambade and Mandrekar 2012). Researchers have established that the brain's cellular OS level contributes to neuronal functions and neurotransmissions (Ali, Al, et al. 2021). Oxidative stress is strongly linked to chronic inflammation, neuroinflammation, and recurring infections in animals with an imbalanced redox system. The neuronal function may be improved through an antioxidant treatment that reduces ROS generation and changes redox‐related signaling pathways (Adnan, Chy, Kamal, et al. 2020). In plants, phenolic compounds act as antioxidants and can improve depression and anxiety conditions by scavenging reactive oxygen species (ROS) and inhibiting inflammation signaling systems (Winiarska‐Mieczan et al. 2020; Chen et al. 2021). It is believed that polyphenols have potential health benefits as antioxidants. Polyphenols are secondary metabolites found in plants. They can boost neuronal energy by scavenging ROS and suppressing inflammation signaling, thereby alleviating depression and anxiety symptoms (Pandey and Rizvi 2009; Wang et al. 2017). What is more, in the central nervous system, flavonoids suppress neuroinflammation, activate GABA_A_‐Cl channels, modify monoaminergic neurotransmitters, and enhance dopamine, serotonin, and monoamine levels (Ali, Sayem, et al. 2021; Sarkar et al. 2022). In our present study, a moderate amount of phenolic and flavonoid contents was found in MOIB (315.21 ± 0.39 mg GAE/g dry extract and 308.80 ± 1.49 mg QE/g dry extract, respectively). These values are comparable to those reported in earlier studies on *Allophylus villosus* and *Mycetia sinensis*, such as by Azam et al. (2025), who also observed substantial levels of phenolic and flavonoid compounds, reinforcing the plant's potential antioxidant capacity (Azam et al. 2025). In addition, there is evidence that oxidative stress contributes to anxiety‐related behavior by disrupting neurotransmission and brain function (Bouayed and Soulimani 2019). Through a variety of processes such as single oxygen transfer, hydrogen transfer, lipoxygenase inactivation, and chelation of peroxidase metals, antioxidants can prevent or delay substrate oxidation (Zeb 2020). In the DPPH assay, MOIB showed a concentration‐dependent antioxidant activity with an IC_50_ of 19.67 μg/mL, comparable to ascorbic acid. This aligns with previous reports on *Hygrophila spinosa*, confirming its strong free radical scavenging potential (Uddin et al. 2024). Furthermore, during redox processes, substances with reducing potential can transfer electrons, converting free radicals into less reactive molecules. However, buffer systems can boost antioxidant activity by enhancing the ratio between protonated and deprotonated antioxidants. Several studies have examined how polyphenol structure and ferric reduction capacity are related. In the reducing power assay, MOIB exhibited a maximum absorbance of 0.755 at the highest tested concentration (500 μg/mL), indicating strong electron‐donating ability. This finding is in agreement with earlier studies on *Crotalaria quinquefolia* , which also demonstrated significant reducing activity linked to its rich polyphenolic content (e.g., Hasan Zilani et al. 2024). All of the aforementioned results indicate that MOIB may be considered as a potential therapeutic option for treating oxidative stress, anxiety, and depression.

In terms of in vivo study, two methods, namely, EPM and HBT were used to evaluate anxiolytic activity (Karim et al. 2015). In the EPM test, more time spent along with an elevated number of entries in open arms of the EPM device is thought to reduce anxiety (Ali, Al, et al. 2021). It was noticed that both doses (200 and 400 mg/kg) of MOIB significantly (*p < 0.5, **p < 0.01, and ***p < 0.001) augmented the time spent as well as several entries in open arms as illustrated in Figure 4A,B. This outcome is consistent with earlier findings that highlight the anxiolytic potential of *Crotalaria quinquefolia* extracts in preclinical models (Hasan Zilani et al. 2024). Additionally, apart from anxiolytic activity, HBT was also used to assess mouse exploratory behaviors as well as several aspects of unconditioned behavior during an unknown environment (Thoeringer et al. 2010). Here, anxiolytic activity is indicated by more head dipping (hole poking), while anxiety is indicated by hesitation to poke the hole (Sillaber et al. 2008). In this case, compared to diazepam, MOIB produced a substantial upsurge in head dipping at the higher dose depicted in Figure 5. Therefore, the anxiolytic properties of MOIB may be associated with the activation of GABA‐activated chloride channels, which enhance GABA receptor sensitivity.

On the other hand, locomotor activity has been considered an important tool in evaluating the effect of crude extracts on the central nervous system. A decline in locomotion in an unfamiliar environment indicates a CNS depressive effect. There have been numerous widely accepted methods for evaluating locomotor activity, including OFT and HCT (Geier and Luna 2009). The administration of MOIB at 400 mg/kg significantly decreased locomotor activity, similar to standard at 90 and 120 min (*p < 0.5, **p < 0.01, and ***p < 0.001) both in OFT and HCT, indicating CNS depressant activity (Figures 6 and 7, respectively). This effect may be linked to the GABAergic inhibition in the CNS through membrane hyperpolarization, as GABA and glutamate are the dominant inhibitory neurotransmitters in the brain that lower neural firing rates because of the prolongation of the GABA‐gated channel opening period or an increase in GABA affinity.

To determine whether the mice were in a depressive state, the forced swimming test (FST) as well as the tail suspension test (TST) were used (Ishola et al. 2012). The decrease in immobility time at the highest dose of MOIB revealed significant antidepressant potential in both FST and TST (Figures 8 and 9, respectively) comparable to that of imipramine, which inhibits serotonin and norepinephrine reuptake, and it is plausible to speculate about a similar mechanism of action for MOIB. Additionally, it has been found that appropriate antidepressant function is controlled by an increase in noradrenaline and serotonergic transmission in the brain, which supports the plant's apoptogenic properties, reduces numerous stress parameters, and enhances monoaminergic activity (Kawaura et al. 2009).

To identify drug candidates from multifarious sources, computer‐aided drug design (CADD) has become a buzzword across the world (Gurung et al. 2021; Siddiquee et al. 2024). In this regard, ADMET profiling is a great screening technique for examining the molecular weight, lipophilicity, hydrogen acceptors, hydrogen donors, and different types of toxicity of the compounds, which are crucial for predicting the suitability of the compounds to be drug candidates. The seven compounds exhibited drug‐like properties and good GI absorption and they were also able to cross blood–brain barrier, which is an important feature of neurological drugs (Table 3). Moreover, none of the compounds violated the Lipinski rules and Veber rules. It has been reported that animal toxicity tests are not only costly and time‐consuming but also harmful to the body. In this test, all 7 bioactive compounds showed satisfactory toxicity levels such as hepatotoxicity AMES toxicity, and so on (Table 4).

Molecular docking is a crucial technique in computer‐aided drug design, enabling virtual screening of compound libraries to identify protein‐ligand complexes with the best binding affinity and least energy (Islam, Aktaruzzaman, Barai, et al. 2025; Islam, Aktaruzzaman, Saif, et al. 2025). For all of the targeted receptors, the docking analysis showed a good binding affinity for (CID 550198) and (CID 10393) compared to that of standards (Diazepam and imipramine) (Table 5). Moreover, these two compounds interacted with the targeted receptors through the formation of different bonds, including hydrogen bonds, polar bonds, and hydrophobic bonds (Table 6). Additionally, there were some common interacting residues found both in our proposed compounds and standard drugs that demonstrate the possibility of those compounds as natural drug candidates. These findings are consistent with previous studies that have reported similar phytochemicals with superior or comparable binding profiles to conventional anxiolytic and antidepressant agents (Uddin et al. 2024). This suggests that the selected compounds may serve as promising natural alternatives for managing anxiety and depression through receptor‐specific interactions.

Molecular dynamics simulations have become an essential tool for computer‐aided design, which analyzes the movements of atoms. The Cα‐RMSD explains the overall stability of the proteins and their complexes in a biological system, where higher values indicate more significant deviations from the starting structure. Collectively, these results underscore the stability and low fluctuations of protein‐ligand complexes (Figure 13). Besides, the RMSF trajectory analysis provides valuable insights into the flexibility and dynamic behavior of the protein‐ligand complexes. An RMSF with a higher value indicates a more flexible residue, whereas an RMSF with a lower value indicates a more stable system (Sadr et al. 2021). In this study, two compounds (CID 550198 and CID 10393) showed promising RMSF values that indicate the firm attachment to the target protein binding pockets (Figure 14). These results align with earlier molecular dynamics studies (Hasan Zilani et al. 2024), which have shown that decreased RMSF values are indicative of greater binding stability and limited structural fluctuations at the protein‐ligand interaction site. In addition, RG analysis demonstrates the overall compactness and conformational stability of protein‐ligand complexes. The lower RG value indicates a high level of compactness, and the larger value indicates a dissociation of the compounds from the protein (Samad et al. 2022). Interestingly, all complexes demonstrated steady trajectories throughout the simulations, suggesting consistent maintenance of structural compactness. Again, higher SASA values indicate less stable structures, whereas lower values indicate tightly contracted complexes of water molecules and amino acids. In this study, SASA values of our selected compounds indicated higher stability (Figure 15).

PCA from the MD simulations provides a comprehensive understanding of the distinct influences that different ligands exert on the modes of motion within their respective protein complexes (David and Jacobs 2014; Saif et al. 2025). The 4UUJ complexes demonstrated a balanced distribution of motion, with both PC1 and PC2 accounting for significant variances, triggering considerable structural rearrangements (Figure 16). These findings are similar to previous PCA‐based studies (Hosen et al. 2023), which reported that balanced PC1 and PC2 contributions reflect coordinated domain motions in ligand‐bound states. The 6X3X complexes and ligands continue to drive diverse behaviors. The 6X3X‐CID550198 and CID10393 complexes exhibited a dominant shift primarily along PC1, implying a pronounced ligand‐induced structural modulation and a profound effect of this ligand. Then, the 6X3X‐Diazepam complex had a milder PC1 influence, which demonstrated a subtler mode of motion. Again, the 5I6X‐CID550198 complex experienced a moderate variance shift, while the 5I6X‐CID10393 complex displayed substantial conformational changes driven by its predominant PC1. Lastly, the 5I6X‐Imipramine complex's dominant PC1 underlines significant ligand‐specific structural dynamics.

## 5 Conclusion

The present study demonstrates that *O. indicum* holds significant therapeutic potential, particularly for the treatment of sleep, anxiety, and depressive disorders. Phytochemical screening confirmed the presence of bioactive compounds with anxiolytic and antidepressant properties, supporting its traditional use in folklore medicine. Moreover, *in silico* evaluations revealed that several phytoconstituents exhibit strong binding affinities, along with favorable pharmacokinetic and safety profiles, underscoring their potential as lead compounds for drug development. These findings suggest that *O. indicum* is a promising candidate for drug development. However, the isolation, purification, and in‐depth biological evaluation of specific active constituents are necessary to elucidate the mechanisms underlying its pharmacological effects.

## Author Contributions

**Md. Aktaruzzaman:** conceptualization (equal), formal analysis (equal), investigation (equal), methodology (equal), resources (equal), software (equal), writing – original draft (equal). **Md. Enamul Kabir Talukder:** data curation (equal), methodology (equal), resources (equal), writing – original draft (equal). **Trina Mitra:** data curation (equal), methodology (equal), software (equal), writing – original draft (equal). **Md. Asibur Rahman:** data curation (equal), methodology (equal), software (equal), writing – original draft (equal). **Md. Tarikul Islam:** data curation (equal), methodology (equal), software (equal), writing – original draft (equal). **Jannatul Ferdous:** visualization (equal), writing – original draft (equal). **Nazmul Hossain:** data curation (equal), formal analysis (equal). **Ahmed Saif:** data curation (equal), formal analysis (equal). **Jonas Ivan Nobre Oliveira:** writing – review and editing (equal). **Md. Obayed Raihan:** writing – review and editing (equal). **Saira Rehman:** writing – review and editing (equal). **Bratati Sikdar:** writing – review and editing (equal). **Kishore Kumar Sarkar:** conceptualization (equal), supervision (equal), writing – review and editing (equal).

## Ethics Statement

The Ethical Review Committee of the Faculty of Biological Science and Technology, Jashore University of Science and Technology, approved all the experiments that were performed as per the recommended guidelines of this committee [Ref: ERC/FBST/JUST/2021–81].

## Conflicts of Interest

The authors declare no conflicts of interest.

## Supporting information


**Table S1:** fsn371391‐sup‐0001‐TableS1.docx.

## Data Availability

It is only the summary of the data that is reported in this article. In case of a reasonable request, datasets are available from the corresponding author.
