# Supplementary material for: Oroxylum indicum (L.) Bark Ameliorates Anxiety and Depression: Evidence From Experimental and Computational Studies
Source: Food Sci Nutr. 2026 Jan 14;14(1):e71391. doi: 10.1002/fsn3.71391 (PMC12800905; doi:10.1002/fsn3.71391)
Supplement: Supplementary file 1 — Table S1: fsn371391‐sup‐0001‐TableS1.docx. [file FSN3-14-e71391-s001.docx]

Table S1: ADME profiling of the phytochemical compounds of essential oil in leaves of *Oroxylum indicum* bark.

| **Lipinski’s Rules** | | | | | | | **Veber Rules** | |
| --- | --- | --- | --- | --- | --- | --- | --- | --- |
| **SI** | **Compounds** | **MW**  **(g/mol)**  **<500** | **HBA**  **<10** | **HBD**  **<5** | **Log P**  **≤5** | **Lipinski’s**  **Violations**  **≤1** |  |  |
|  |  |  |  |  |  |  | **nRB**  **≤10** | **TPSA**  **≤140** |
| 1 | 2,4,4,6,6,8,8-Heptamethyl-1-nonene | 224.43 | 0 | 0 | 5.83 | No | 6 | 0.00 Å² |
| 2 | 1,2,3,4-Tetramethylcyclohexane | 140.27 | 0 | 0 | 3.32 | No | 0 | 0.00 Å² |
| 3 | [Benzeneethanol, 4-hydroxy-](https://www.ncbi.nlm.nih.gov/pcsubstance/?term=%22Benzeneethanol%2C%204-hydroxy-%22%5bCompleteSynonym%5d%20AND%2010393%5bStandardizedCID%5d) | 138.16 | 2 | 2 | 0.93 | No | 2 | 40.46 Å² |
| 4 | [Eicosen-1-ol, cis-9-](https://www.ncbi.nlm.nih.gov/pcsubstance/?term=%22Eicosen-1-ol%2C%20cis-9-%22%5bCompleteSynonym%5d%20AND%205364523%5bStandardizedCID%5d) | 296.53 | 1 | 1 | 6.80 | No | 17 | 20.23 Å² |
| 5 | 9-OCTADECENOIC ACID, (E)- / trans-9-Octadecenoic acid | 282.46 | 2 | 1 | 6.11 | No | 15 | 37.30 Å² |
| 6 | 2-Methyl-5-undecanol | 186.33 | 1 | 1 | 3.75 | No | 8 | 20.23 Å² |
| 7 | Ethyl 9-hexadecenoate | 282.46 | 2 | 0 | 5.81 | No | 15 | 26.30 Å² |
| 8 | Carbonic acid, decyl 2-ethylhexyl ester | 314.50 | 3 | 0 | 6.50 | Yes | 17 | 35.53 Å² |
| 9 | Dicyclomine | 309.49 | 3 | 0 | 4.40 | No | 8 | 29.54 Å² |
| 10 | Clofexamide | 284.78 | 3 | 1 | 2.18 | No | 9 | 41.57 Å² |
| 11 | Ethyl 10-undecenoate | 212.33 | 2 | 0 | 3.86 | No | 11 | 26.30 Å² |
| 12 | 1,7-Dimethyl-4,10-dioxa-1,7-diazacyclododecane | 202.29 | 4 | 0 | -0.86 | No | 0 | 24.94 Å² |
| 13 | 4-(3-Hydroxyphenyl)-4-oxobutanoic acid | 194.18 | 4 | 2 | 1.44 | No | 4 | 74.60 Å² |
| 14 | 1,2,3,4-Tetramethylcyclohexane | 140.27 | 0 | 0 | 3.32 | No | 0 | 0.00 Å² |
| 15 | l-(+)-Ascorbic acid 2,6-dihexadecanoate | 652.94 | 8 | 2 | 10.09 | Yes | 34 | 119.36 Å² |
| 16 | Ethyl 2,4,6-trimethylbenzoate | 192.25 | 2 | 0 | 2.79 | Yes | 3 | 26.30 Å² |
| 17 | Cholestan-3-ol, 2-methylene-, (3.beta.,5.alpha.) | 400.68 | 1 | 1 | 7.63 | No | 5 | 20.23 Å² |
| 18 | [Heptadecanoic acid, 16-methyl-, methyl ester](https://www.ncbi.nlm.nih.gov/pcsubstance/?term=%22Heptadecanoic%20acid%2C%2016-methyl-%2C%20methyl%20ester%22%5bCompleteSynonym%5d%20AND%20110444%5bStandardizedCID%5d) | 298.50 | 2 | 0 | 6.28 | No | 16 | 26.30 Å² |
| 19 | METHYL 10-TRANS,12-CIS-OCTADECADIENOATE | 294.47 | 2 | 0 | 5.97 | No | 15 | 26.30 Å² |
| 20 | 1,3,3-Trimethyl-2-hydroxymethyl-3,3-dimethyl-4-(3-methylbut-2-enyl)-cyclohexene | 222.37 | 1 | 1 | 4.09 | No | 3 | 20.23 Å² |
